# Oxidation State Determines Solvent Structure Around a Manganese–Vanadium Polyoxometalate Water‐Oxidation Catalyst

**DOI:** 10.1002/anie.9168848

**Published:** 2026-04-16

**Authors:** Simon Tippner, Moritz Remmers, Sebastian Mai, Boris Mashtakov, Mihail Mondeshki, Carsten Streb, Leticia González

**Affiliations:** ^1^ Institute of Theoretical Chemistry, Faculty of Chemistry University of Vienna Vienna Austria; ^2^ University of Vienna Doctoral School in Chemistry (DoSChem) Vienna Austria; ^3^ Department of Chemistry Johannes Gutenberg University Mainz, Duesbergweg 10–14 Mainz Germany

**Keywords:** molecular dynamics, polyoxometalates, solvation, water oxidation

## Abstract

Understanding how microsolvation influences the reactivity and stability of molecular water‐oxidation catalysts remains a central challenge in artificial photosynthesis. Here, we combine molecular dynamics (MD) simulations with spectroscopic and electrochemical experiments to elucidate how acetonitrile/water mixtures organize around the mixed‐valence polyoxometalate [(Mn_4_O_4_) (V_4_O_13_) (OAc)_3_]^n−^ across catalytically relevant redox states. Our results reveal a pronounced oxidation‐state dependence: the reduced ${\left\{\mathrm{MnV}\right\}}^{3-}$ species is surrounded by a dense, highly structured hydration shell even at low water contents, preferentially engaging terminal vanadate oxygen sites and partially displacing acetonitrile from the first solvation shell. By contrast, the oxidized ${\left\{\mathrm{MnV}\right\}}^{2-}$ and ${\left\{\mathrm{MnV}\right\}}^{1-}$ species show substantially weaker water structuring and largely oxidation state–insensitive acetonitrile organization. These microscopic solvation motifs are directly reflected experimentally: spectroscopic titrations reveal the emergence of hydrogen‐bond formation at V═O groups and Jahn–Teller–driven asymmetric solvent accumulation accompanied by ligand exchange, while electrochemical measurements indicate reduced diffusion coefficients and diminished redox features at higher water contents, consistent with ion pair–mediated aggregation observed in MD simulations. Together, these results establish oxidation state as a key control parameter for solvent organization around polyoxometallate water‐oxidation catalysts and provide a molecular rationale for the enhanced activity yet limited stability window of ${\left\{\mathrm{MnV}\right\}}^{n-}$ species in acetonitrile/water mixtures.

## Graphical abstract

Oxidation state controls microsolvation in the polyoxometalate ${\left\{\mathrm{MnV}\right\}}^{n-}$ water‐oxidation catalyst in acetonitrile/water mixtures. The reduced species {MnV}^3−^ attracts a highly structured hydration layer even at low water content, preferentially binding terminal V═O sites and excluding acetonitrile, while oxidized species show weaker hydration and stronger aggregation.

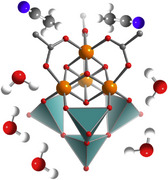

## 1 Introduction

Understanding solvent effects in catalysis [1, 2, 3] is of pivotal importance as they modulate energetics, reaction kinetics and mechanistic pathways [4, 5, 6]. Yet, subtle structural reorganizations and dynamic solvent interactions often elude direct observation so that their effects are typically inferred indirectly from changes in reaction rates or solvent‐dependent variations in product selectivity, rather than being resolved at the molecular level, which restricts rational design of efficient catalysis. In catalytic water splitting [7], water is particularly important as it serves not only as medium but also as reagent to be split into H_2_ and O_2_. The water oxidation step is most challenging, requiring catalysts that can access multiple oxidation states and mediate transfer of four electrons and four protons while remaining stable under harsh oxidative conditions [8, 9]. Nature accomplishes this feat with the Mn_4_CaO_5_ cluster of Photosystem II [10, 11], whose design principles have inspired extensive efforts toward polynuclear molecular water‐oxidation catalysts (WOCs) [8, 12]. In the field of polyoxometalates (POMs), this has fostered the development of numerous transition metal functionalized WOC models including Ru‐, Co‐, and Mn‐based systems [13, 14, 15, 16, 17].

**SCHEME 1:**
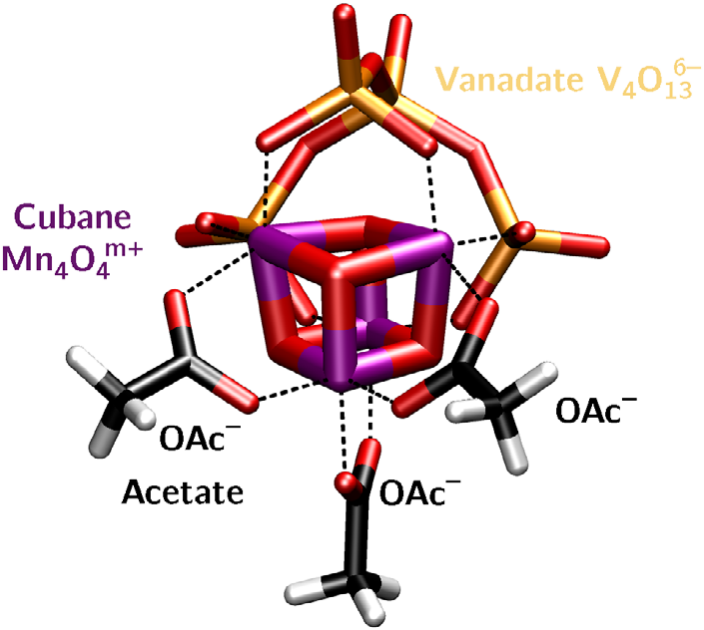
Three‐dimensional depiction of [(Mn_4_O_4_) (V_4_O_13_) (OAc)_3_]^n−^.

As a structural mimic of the natural oxidation complex, herein we focus on the mixed‐valence manganese—vanadium oxide POM [(Mn_4_O_4_) (V_4_O_13_) (OAc)_3_]^n−^, hereafter referred to as ${\left\{\mathrm{MnV}\right\}}^{n-}$, where n denotes the overall anionic charge [18]. It features a central [Mn_4_O_4_]^6+^ cubane core, capped by a [V_4_O_13_]^6−^ vanadate unit, as well as three bridging acetate ligands (Scheme 1). This POM has shown promising water oxidation activity under photochemical and electrochemical conditions, with a maximum turnover frequency of up to $3.6\;s^{-1}$ and maximum turnover numbers as high as 12 000 when operated in acetonitrile/water mixtures; however, it is unstable in pure water, degrading into Mn─V‐oxide colloids [19]. This pronounced solvent dependence indicates that catalytic performance and stability are governed not only by the intrinsic properties of the cluster but also by its solvation environment, underscoring the need for molecular‐level understanding of how solvent structure and composition modulate reactivity and degradation pathways.

Prior theoretical studies have investigated the reactivity of the ${\left\{\mathrm{MnV}\right\}}^{n-}$ cluster without explicitly accounting for solvent‐catalyst interactions [20, 21, 22, 23, 24], even though experiments reveal clear differences in reactivity as a function of the acetonitrile:water ratio [18]. Describing explicit solvent environments is an intrinsic challenge, where oxidation state–dependent charge redistribution, hydrogen bonding, and competitive solvation give rise to dynamic solvent structures that are difficult to probe experimentally and costly to model theoretically beyond implicit models. However, this type of study is essential, as pioneering MD simulations [25] of the Keggin anion [XW_12_O_40_]_n_^−^ (X = $P^{5+}$ (*n* = 3), ${\mathrm{Si}}^{4+}$ (*n* = 4), ${\mathrm{Al}}^{3+}$ (*n* = 5)) in aqueous solvent have revealed that the overall cluster charge has a significant impact on water solvation structure and interactions with counter‐cations (here: Li^+^, Na^+^, K^+^), and were in agreement with comparable experimental data, for example, on diffusion behaviour [26]. In addition, experimental electrochemical studies revealed that solvent reorganization around Keggin‐type POMs changes systematically with the cluster charge, emphasizing direct correlations between redox behaviour and solvent environment [27]. And yet, to our knowledge, no studies have addressed the explicit solvent environment around POM‐WOCs using correlated theory and experiment, despite strong solvent‐dependent effects have been also observed in other molecular WOCs. Meyer and co‐workers found that the rate of water oxidation increased when their Ru‐WOC was operated in nonaqueous solvents containing water as the reagent, and even observed the emergence of new reaction pathways [28, 29]. Further, they demonstrated that the oxidation activity of a related Ru‐WOC was highly dependent on the proton content of the aqueous solution [30]. Bernasconi et al. suggested that the water‐shell around an Fe‐WOC played a key role in proton management during the water oxidation cycle [31]. Similarly, Yang and coworkers showed that intramolecular hydrogen bonding was a key factor in the reactivity of Co‐WOC complexes [32]. Also for heterogeneous electrochemical WOC at IrO_2_ nanoparticles operated in acidified acetonitrile‐water mixtures, Girault and colleagues identified a “tipping point,” at which low water concentrations lead to a decrease of the WOC onset potential. This unusual behavior was linked to the disruption of the hydrogen‐bond network between water molecules, resulting in the formation of isolated water clusters, which are more easily oxidized compared to bulk water [33]. Despite the chemistry and reactivity of POM anions is often modulated or even controlled by their counter‐cations [34], efforts to rationalize the impact of the solvent environment on catalytic activity remain scarce. In a pioneering study, Nazmutdinov and co‐workers used density functional theory (DFT) and MD simulations to explore the stability and reactivity of a prototype Co‐POM‐WOC, showing that in aqueous solution, an Na^+^‐mediated water shell is formed near the POM cluster [35], resulting in contact‐ion pairs which are suggested to facilitate electron transfer steps during the water oxidation [32]. Related experimental studies revealed the impact of aqueous electrolyte composition and pH value on the stability and reactivity of this POM‐WOC [36]. These studies highlight that a combination of theoretical simulations and complementary (in situ) experimental studies can shed light on complex POM‐WOC speciation patterns in solution. This is a prerequisite to understand the true nature of dynamic catalysts under operation [13].

Here, we show for the first time that the solvent environment decisively governs the redox chemistry, reactivity, and stability of the MnV POM operated in acetonitrile/water mixtures. To unravel these solvent‐driven effects, we combine MD simulations with spectroscopic and electrochemical experiments, providing a unified, molecular level choreography of how acetonitrile and water organize around the catalyst in different oxidation states. Explicit resolution of solvent–catalyst interactions reveals, with atomic resolution, how solvation shells form and reorganize upon redox changes, and how these structural responses directly control the stability, reactivity, and degradation into colloids of POM‐based WOCs. Broadly, our findings provide deeper atomistic insight into the intrinsic correlation of microsolvation and redox chemistry, with implications in catalysis.

## 2 Results and Discussion

Earlier work [20, 21, 22, 23, 24] has shown that the catalytically active species ${\left\{\mathrm{MnV}\right\}}^{1-}$ (which contains the four Mn atoms in a formal oxidation state IV, i.e., ${\mathrm{Mn}}_{4}^{\text{IV}}$) is formed after two oxidation steps from the precatalyst ${\left\{\mathrm{MnV}\right\}}^{3-}$ (${\mathrm{Mn}}_{2}^{\text{III}}\;{\mathrm{Mn}}_{2}^{\text{IV}}$) via the ${\left\{\mathrm{MnV}\right\}}^{2-}$ (${\mathrm{Mn}}^{\text{III}}\;{\mathrm{Mn}}_{3}^{\text{IV}}$) configuration. Therefore, computations need to be done for the three different oxidation states, from which only ${\left\{\mathrm{MnV}\right\}}^{3-}$ and ${\left\{\mathrm{MnV}\right\}}^{2-}$ are experimentally accessible, while the two‐electron oxidized species ${\left\{\mathrm{MnV}\right\}}^{1-}$ is difficult to stabilize and isolate experimentally [20]. Note that the catalyst is only stable in acetonitrile solutions containing 0–5 vol% water [18]. However, as a reference standpoint, we first analyze the solute–solvent interactions in pure solvents (water and acetonitrile), before turning to the mixed system. Accordingly, for each of the three oxidation states (${\left\{\mathrm{MnV}\right\}}^{3-}$, ${\left\{\mathrm{MnV}\right\}}^{2-}$, and ${\left\{\mathrm{MnV}\right\}}^{1-}$), three sets of MD simulations were carried out during 200 ns: in pure water, acetonitrile, and in their mixture. Details of the MD simulations are in Section S1 of the Supporting Information. Tetrabutylammonium (${\left[{\mathrm{NBu}}_{4}\right]}^{+}$) was used as a counterion. Additionally, MD simulations were performed with five POMs in the two experimentally accessible charged states, ${\left\{\mathrm{MnV}\right\}}^{3-}$ and ${\left\{\mathrm{MnV}\right\}}^{2-}$, to investigate aggregation. Force fields for the POM in its three oxidation states were newly parametrized (Section S1). The atom type definitions and some angle parameters were taken from ref. [21]. Bond stretch parameters were revised to obey symmetry and reproduce the bond length distributions of a quantum mechanics/molecular mechanics reference trajectory. All dihedral parameters involving two Mn atoms were set to zero because the (Mn_4_O_4_)(V_4_O_13_) cage is already rigid using only angle potentials; hence, removing the redundant dihedral parameters reduces artificial strain and allows easier parametrization. Dihedral potentials involving V and C atoms were kept to avoid twisting of the V─O─V bridges and deplanarization of the acetates. Furthermore, whereas ref. [21] used Mulliken charges for the atomic partial charges, in the revised force field the CHELPG procedure [37] was used to more faithfully represent the electrostatic potential of the molecule [38]. The solvent‐mixture force field was validated through a benchmark study in which several parameters for water and acetonitrile were tested (Section S2). For each set, MD simulations were used to compute mass densities across different solvent compositions, which were then compared with experimental reference values.

### 2.1 Simulations of ${\left\{\mathrm{MnV}\right\}}^{n-}$ in Pure Solvents

**FIGURE 1:**
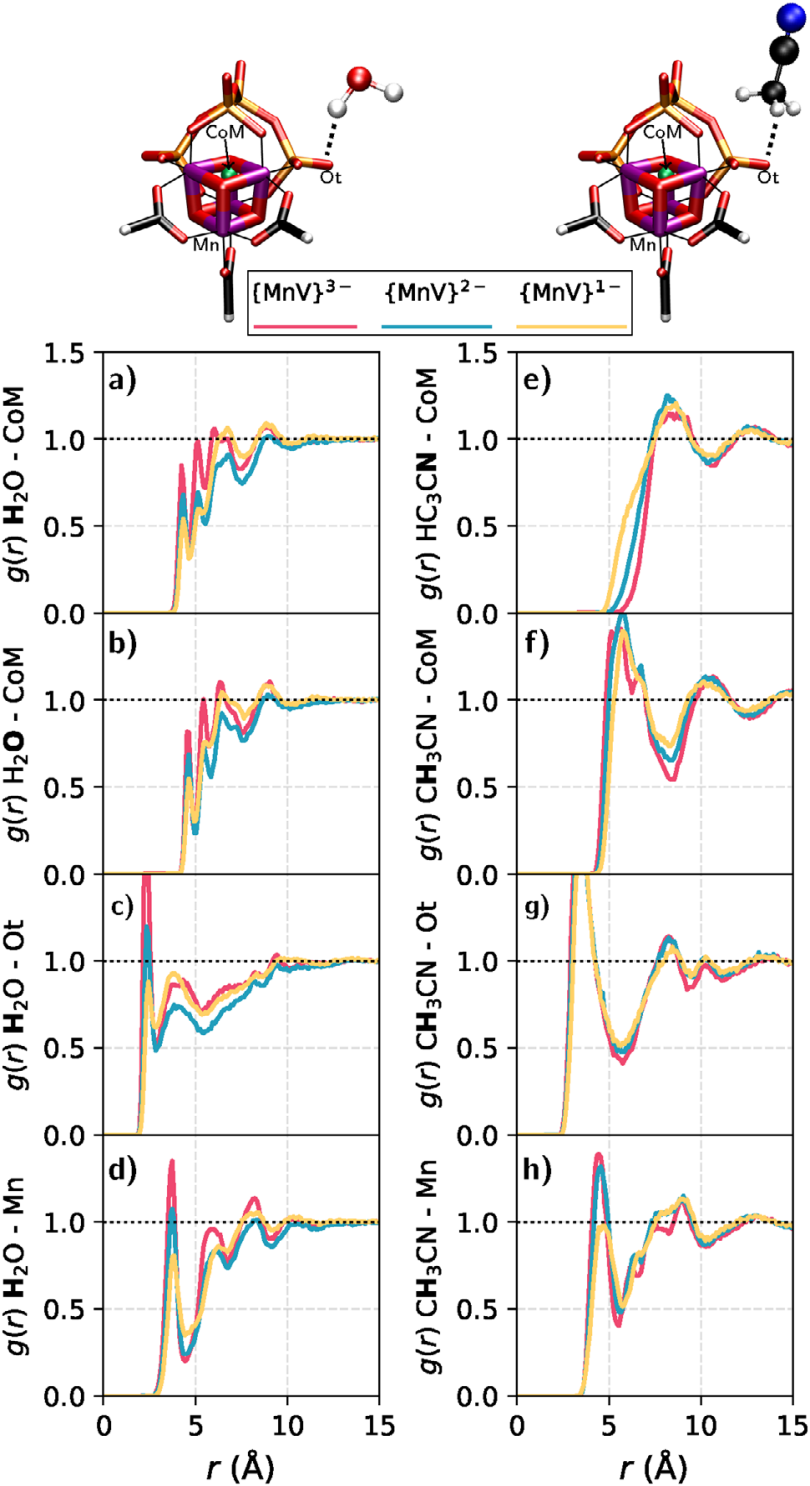
Radial distribution functions, g(r), of the ${\left\{\mathrm{MnV}\right\}}^{n-}$ in pure water (left) and pure acetonitrile (right). Functions are plotted between specific atoms of the solvent and either (panels a,b,e,f) the center of mass (CoM) of the cluster, or (c,g) the terminal oxygen of the cluster, denoted Ot (OC1 in Figure S4) or (d,h) the apical Mn atom of the cubane (Mn2 in Figure S4). For illustration, a green dummy atom is placed at the CoM of the ${\left\{\mathrm{MnV}\right\}}^{n-}$ cluster. Three‐dimensional renderings of ${\left\{\mathrm{MnV}\right\}}^{n-}$ including an example of the most frequent solvent molecule were generated with VMD [39].

To probe solvent organization around the catalyst, we computed radial distribution functions (RDFs) from MD simulations of ${\left\{\mathrm{MnV}\right\}}^{3-}$, ${\left\{\mathrm{MnV}\right\}}^{2-}$, and ${\left\{\mathrm{MnV}\right\}}^{1-}$ dissolved in pure water (Figure 1a–d) and pure acetonitrile (Figure 1e–h). RDFs, which are proportional to the probability of finding two particles at a particular distance, reveal solvation shells and pinpoint hydrogen bonds.

The increasing ion–dipole interactions between the cluster anion and the water molecules is reflected in the intensity of the RDFs, which increases with the negative charge of the POM (${\left\{\mathrm{MnV}\right\}}^{1-}\to {\left\{\mathrm{MnV}\right\}}^{2-}\to {\left\{\mathrm{MnV}\right\}}^{3-}$), see Figure 1a–d. The analysis of the RDFs between the center of mass (CoM) and both oxygen and hydrogen atoms of water provides insight into the average orientation of water molecules relative to the catalyst. The shorter distances for the H atoms compared with the O atoms indicate that water forms hydrogen bonds with the hydrogen atoms pointing to ${\left\{\mathrm{MnV}\right\}}^{n-}$. More detailed local solvation patterns are resolved computing site‐specific RDFs between the hydrogens of water or acetonitrile and each of the 23 oxygen atoms of the catalyst, as well as the four manganese atoms (Section S3). Two representative cases, the terminal oxygen site (Ot) and the apical Mn atom are depicted in panels 1c,d. The largest RDF intensities occur for Ot, indicating a preferential accumulation of water around the vanadate ligand ${\left[V_{4}O_{13}\right]}^{6-}$. This trend is further supported by similar high intensities for other terminal oxygen sites (Figure S4) and becomes even more pronounced upon reduction to ${\left\{\mathrm{MnV}\right\}}^{3-}$, showcasing the strong influence of electrostatic effects. In contrast, the apical Mn site shows weaker interactions, as the vanadate cap and acetate groups block access from that side.

In pure acetonitrile, the CN group is oriented away from ${\left\{\mathrm{MnV}\right\}}^{n-}$. The methyl hydrogens approach the cluster more closely than the nitrogen atom, as shown by shorter CoM–H distances (Figure 1e) compared with the CoM‐N distances (Figure 1f). This orientation is expected from ion‐dipole interactions and so the polar CN group points away from the negatively charged ${\left\{\mathrm{MnV}\right\}}^{n-}$, while the methyl group points toward it. As in water, the largest RDF intensities are found for Ot (Section S3), showing that both solvents preferentially accumulate at the vanadate ligand due to its high charge density. However, the first RDF peak appears at longer distances in acetonitrile than in water, indicating that water molecules can approach the catalyst closer, likely due to the larger steric bulk of acetonitrile and ability to form hydrogen bonds. The two representative cases are shown in Figure 1g,h. It is interesting that while water responded strongly to changes in the oxidation state, showing significantly increased peak intensities for more negatively charged species, acetonitrile is far less sensitive due to its nonprotic character.

The analysis of integrated solvation numbers (denoted as N(r) in Section S4) shows that the first peak of the RDFs corresponds to a single solvent molecule. Beyond this first shell, cluster‐solvent effects extend up to distances of ca. 10 Å above which the solvation numbers of the respective charge states converge, indicating a transition to bulk solvent. Consistent with this behavior, enhanced solvent structuring extends to 10 Å in water and 15 Å in acetonitrile, beyond which the RDFs converge to bulk‐like behavior.

### 2.2 Simulations of ${\left\{\mathrm{MnV}\right\}}^{n-}$ in Acetonitrile/Water Mixtures

**FIGURE 2:**
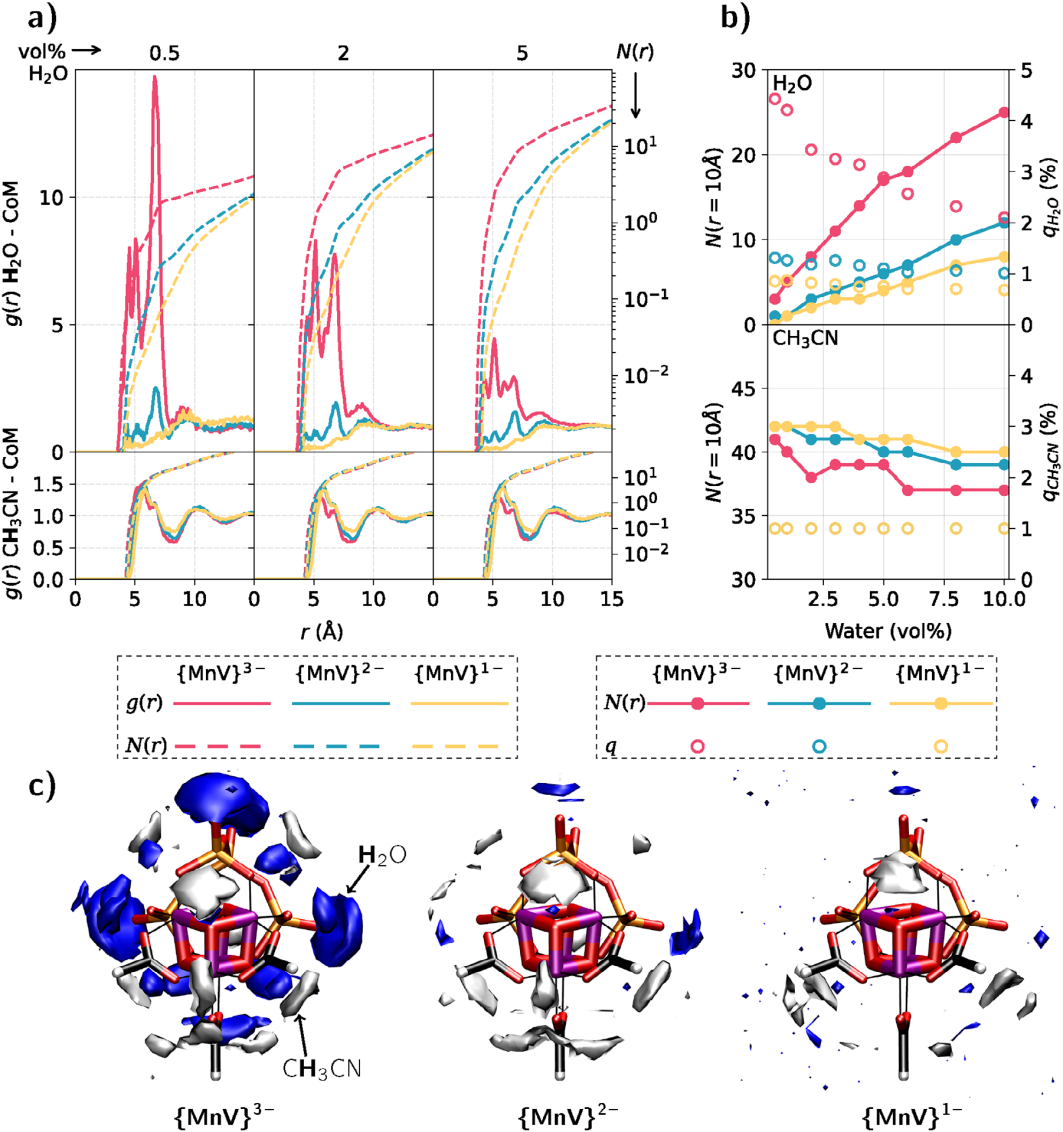
(a) Radial distribution functions, g(r), between the center of mass (CoM) of the cluster and the hydrogen atoms of water and acetonitrile in solid lines; dashed lines indicate the corresponding integrated solvation numbers N(r). (b) Integrated solvation numbers N(r) at r = 10 Å, are shown as solid lines with filled markers at the data points, while the solvent fraction q (fraction of solvent molecules residing within 10 Å of the catalyst) is shown as hollow circular markers. Both quantities are plotted as a function of water content. (c) 3D‐SDFs at 5 vol% water show solvent organization around the catalyst, with hydrogen atoms of water shown in blue and those of acetonitrile shown in white. The visual cutoffs were chosen as multiples of the expected bulk occupancy of the corresponding hydrogen atoms [40]. For water hydrogens, we used 20 times the expected value for ${\left\{\mathrm{MnV}\right\}}^{3-}$ and ${\left\{\mathrm{MnV}\right\}}^{2-}$, and 4 times for ${\left\{\mathrm{MnV}\right\}}^{1-}$. For acetonitrile hydrogens, a cutoff of four times the expected value was used for all oxidation states.

We now analyze the effects of acetonitrile/water mixtures on the POM using different volumetric percentages of water (Figure 2). As observed in pure water, increasing the anionic charge of ${\left\{\mathrm{MnV}\right\}}^{n-}$ from −1 to −3 leads to a systematic increase in the intensity of the RDFs between water hydrogen atoms and the CoM of the POM. For example (Figure 2a), at 0.5 vol% H_2_O, the first peak at 4.55 Å has a value of 8.01 for ${\left\{\mathrm{MnV}\right\}}^{3-}$, 0.54 for ${\left\{\mathrm{MnV}\right\}}^{2-}$, and 0.53 for ${\left\{\mathrm{MnV}\right\}}^{1-}$, concomitant to the increase in negative charge. However, the nonlinear trend is noteworthy: both ${\left\{\mathrm{MnV}\right\}}^{1-}$ and ${\left\{\mathrm{MnV}\right\}}^{2-}$ show weak solvent fine structuring, while ${\left\{\mathrm{MnV}\right\}}^{3-}$ exhibits a strong interaction with water. This increased intensity points to the formation of a water solvation shell around ${\left\{\mathrm{MnV}\right\}}^{3-}$—supported by the increase in integrated solvation numbers, N(r), upon reduction. As the water content increases, the RDF intensity decreases, which is a consequence of normalization with respect to the bulk density. Notably, between 3 and 5 vol% water, no change in the RDF of ${\left\{\mathrm{MnV}\right\}}^{3-}$ is observed, indicating that the hydration shell might have reached saturation. In contrast, ${\left\{\mathrm{MnV}\right\}}^{2-}$ and ${\left\{\mathrm{MnV}\right\}}^{1-}$ show only minor changes with increasing water content, indicating that their solvation behavior is largely independent of water concentration within the cluster stability range.

In contrast to water, the RDFs involving acetonitrile show no significant change with increasing water content, irrespective of the cluster charge, indicating that the local structuring of acetonitrile is largely insensitive to the cluster charge. Only if zooming in, a decrease in RDF intensity for ${\left\{\mathrm{MnV}\right\}}^{3-}$ compared to ${\left\{\mathrm{MnV}\right\}}^{2-}$ is observed for 5 vol% water, due to the strong interactions between water and ${\left\{\mathrm{MnV}\right\}}^{3-}$, which disrupts the local structuring of acetonitrile. The weak influence of the POM charge on acetonitrile solvation is also reflected in the coordination numbers, N(r), which also exhibit no significant change upon reduction. RDFs between the solvent hydrogen atoms and the reference oxygen and manganese atoms of the catalyst are shown in Section S5. Overall, both water and acetonitrile show preferred accumulation around the terminal oxygen sites, consistent with the trends observed in pure solvent systems.

To quantify how strongly oxidation perturbs its surrounding solvent structure, we evaluated N(r) at r=10 Å and defined the fraction $q_{\mathrm{solvent}}=N(r)/N_{\mathrm{solvent}}$, where $N_{\text{solvent}}$ is the total number of solvent molecules (Figure 2b). The quantity $q_{\mathrm{solvent}}$ measures the fraction of the solvent population located within 10 Å of the catalyst; larger values therefore indicate stronger local solvent accumulation relative to the total solvent content of the simulation box. The cutoff of 10 Å was chosen since beyond this distance the RDFs converge to unity, indicating that we are entering the solvent bulk regime.

For water, N(r = 10 Å) increases steeply for ${\left\{\mathrm{MnV}\right\}}^{3-}$, showing the formation of a dense hydration shell. In contrast, ${\left\{\mathrm{MnV}\right\}}^{2-}$ and ${\left\{\mathrm{MnV}\right\}}^{1-}$ display a notably slower increase. In the fraction 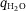, we see that for ${\left\{\mathrm{MnV}\right\}}^{2-}$ and ${\left\{\mathrm{MnV}\right\}}^{1-}$ no significant change is observed, indicating that the increase in N(r=10 Å) is a consequence of the addition of solvent molecules, rather than a preferred accumulation around the catalyst. By contrast, ${\left\{\mathrm{MnV}\right\}}^{3-}$ shows an initial high fraction q, indicating the immediate accumulation of water molecules around the catalyst even at low water contents. Then it decreases, reaching similar values for 3 or 5 vol%, possible due to saturation of the solvation shell.

As discussed above, the behavior of acetonitrile differs from that of water, and indeed it shows similar N(r=10 Å) values under all conditions, with approximately 40 acetonitrile molecules located within 10 Å of the catalyst. For ${\left\{\mathrm{MnV}\right\}}^{3-}$, slightly lower $q_{CH_{3}CN}$ values are observed, consistent with the displacement of acetonitrile by the formation of a strong structured water solvation shell. Note that upon addition of water, the number of acetonitrile molecules near the cluster N(r=10 Å) slightly decreases for all cluster oxidation states, implying replacement by water in each case. The fraction of $q_{{\text{CH}}_{3}\text{CN}}$ remains unchanged upon addition of water and is consistent across all oxidation states, indicating that acetonitrile‐catalyst interactions are independent of both water content and catalyst oxidation state. This behavior reflects the dominance of water in these solvent mixtures. Figure S9 extends this analysis to 99.5, 99, 98, 97, 96, and 95 vol% water, confirming that acetonitrile solvation is minimally affected by the solvent mixture composition.

A better visualization of the local solvent environment around the catalyst in its different charge states is achieved with three‐dimensional spatial distribution functions (3D‐SDF), shown in Figure 2c for 5 vol% water. Such representations also clearly identify oxygen sites involved in solvent interactions. It is remarkable that for ${\left\{\mathrm{MnV}\right\}}^{3-}$, water exhibits the strongest interactions with the terminal oxygen atoms, forming a well‐defined solvation structure. Acetonitrile hydrogen atoms are predominantly located in the remaining pockets not occupied by water. Given that the water distribution is plotted at 20 times its expected bulk value, while acetonitrile is shown at only 4 times its expected bulk value, the 3D‐SDFs illustrate the significantly stronger interactions between water and ${\left\{\mathrm{MnV}\right\}}^{3-}$ than with acetonitrile. Upon oxidation to ${\left\{\mathrm{MnV}\right\}}^{2-}$, water‐cluster interactions are substantially weaker, with acetonitrile weakly structured due to its low sensitivity to the charge. Finally, for ${\left\{\mathrm{MnV}\right\}}^{1-}$, no pronounced fine structure of water around the catalyst is observed; instead, acetonitrile dominates the microsolvation of the catalysts.

Deeper insight into the interactions between the catalyst and water can be obtained calculating the number of hydrogen bonds formed at each oxygen site of the catalyst (Section S6). We found that regardless of charge, most hydrogen bonds are located at the terminal oxygen sites because these sites are most frequently surrounded by solvent water molecules. Further, the number of hydrogen bonds formed does not differ significantly across the individual terminal oxygen sites of the cluster.

In summary, we found that aqueous microsolvation is highly sensitive to the catalyst's oxidation state. Hydration becomes less pronounced upon oxidation from ${\left\{\mathrm{MnV}\right\}}^{3-}$ → ${\left\{\mathrm{MnV}\right\}}^{2-}$ → ${\left\{\mathrm{MnV}\right\}}^{1-}$, while the corresponding effect in acetonitrile is substantially weaker. The enhanced water solvation shells have direct implications for the catalytic water oxidation, as they preorganize the catalyst for the subsequent activation steps. Stronger solvation stabilizes the intermediate that facilitates the subsequent exchange of one acetate ligand by two water molecules [21] and modulates the redox potential of the catalyst, easing charge transfer. As it will be evidenced later, the weak catalyst–water interactions in the catalytic active species ${\left\{\mathrm{MnV}\right\}}^{2-}$ is responsible for aggregation, which ultimately leads to diminished catalytic performance. To obtain experimental evidence supporting our theoretical interpretation of the oxidation state dependency of water microsolvation, we performed electrochemical analyses, Fourier‐transform infrared spectroscopy (FTIR), electrospray ionization mass spectrometry (ESI‐MS), NMR relaxation measurements, and UV–Vis in varying acetonitrile/water mixtures. Details on the used instrumentation can be found in Section S7.

### 2.3 Spectroscopic and Electrochemical Studies

In earlier work [41], we showed that the characteristic manganese d–d optical transitions can be used as a sensitive probe to gain insights into local structure changes at or around ${\left\{\mathrm{MnV}\right\}}^{n-}$. Thus, here we performed an experimental titration study to probe cluster–solvent interactions in acetonitrile containing 0–5 vol% water, which is the stability range for ${\left\{\mathrm{MnV}\right\}}^{3-}$ and ${\left\{\mathrm{MnV}\right\}}^{2-}$ [20]. The ${\left\{\mathrm{MnV}\right\}}^{2-}$ was quantitatively prepared from ${\left\{\mathrm{MnV}\right\}}^{3-}$ by bulk electrolysis [18]. Both species were fully characterized electrochemically (Sections S8–S10). Since ${\left\{\mathrm{MnV}\right\}}^{1-}$ cannot be prepared and stabilized under the given experimental conditions, it was excluded from the study.

**FIGURE 3:**
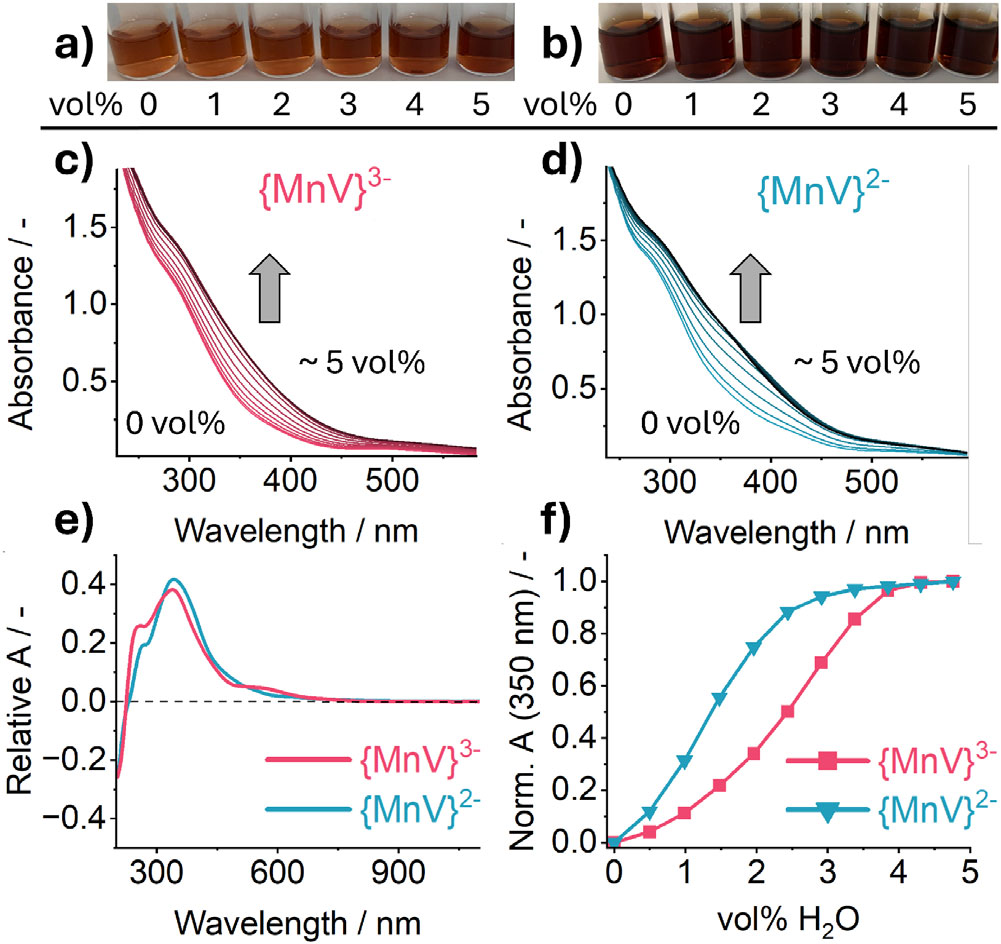
(a,b) The increasing absorbance with addition of water is visible by the darker color of samples prepared at 1 mM concentration of the cluster and gradually increasing vol% water. (c) UV‐Vis spectra of ${\left\{\mathrm{MnV}\right\}}^{3-}$ and (d) of ${\left\{\mathrm{MnV}\right\}}^{2-}$ at a cluster concentration of about 50 μM. Water was added in 20 μL steps to 2 mL of water free sample and the spectra were corrected by the dilution factor. (e) The relative absorbance of the UV‐Vis spectrum at 5 vol% of water after subtraction of the spectrum at 0 vol% of water for ${\left\{\mathrm{MnV}\right\}}^{3-}$ and ${\left\{\mathrm{MnV}\right\}}^{2-}$ and (f) the changes of the normalized absorbance at 350 nm with increasing vol% water of ${\left\{\mathrm{MnV}\right\}}^{3-}$ and ${\left\{\mathrm{MnV}\right\}}^{2-}$.

As shown in Figure 3a,b, adding water to the cluster solution induces a color change from light orange to a more intense brown. UV–Vis measurements (Figure 3c,d and Section S8) confirmed an overall increase in absorption, while the positions of the absorption maxima remained essentially unchanged. Notably, these characteristic spectral changes are distinct from previously reported changes due to cluster protonation or cluster oxidation state changes [18, 41]. The absorption bands can be assigned to the manganese centered d–d transitions of ${\mathrm{Mn}}^{4+}$ at 350 nm, and ${\mathrm{Mn}}^{3+}$ at 600 nm. Figure 3e shows the difference spectra (relative absorbance) of samples containing 5 vol% water compared to the water‐free samples. The increase at approximately 350 nm is more pronounced for ${\left\{\mathrm{MnV}\right\}}^{2-}$ due to the higher number of ${\mathrm{Mn}}^{4+}$ centers. At the same time ${\left\{\mathrm{MnV}\right\}}^{3-}$ shows a more pronounced increase at higher wavelengths, which is attributed to low intensity, Laporte‐forbidden ${\mathrm{Mn}}^{3+}$ d–d transitions [41]. These distinct changes are explained by hydrogen bonding of unsymmetrically distributed water molecules in the solvation shell of the cluster. Breaking the octahedral symmetry through asymmetric vibrations in the presence of hydrogen bonded water molecules can lead to a relaxation of the LaPorte rule, thereby increasing the observed absorbance. Notably, these changes occur earlier for ${\left\{\mathrm{MnV}\right\}}^{2-}$ at water contents up to 3 vol% while ${\left\{\mathrm{MnV}\right\}}^{3-}$ requires about 5 vol% water for this effect to reach completion (see Figure 3f). This is in line with the MD simulations, which show that the solvation shell of ${\left\{\mathrm{MnV}\right\}}^{3-}$ contains larger amounts of water compared to ${\left\{\mathrm{MnV}\right\}}^{2-}$, due to the stronger anion–dipole interactions between the more negatively charged POM and the solvent. This results in the quick formation of a dense water hydration shell around ${\left\{\mathrm{MnV}\right\}}^{3-}$. Note that typically, d–d‐based transitions are only weakly affected by bulk solvent effects (e.g., polarity changes upon addition of water), while changes of the first manganese coordination sphere, for example, by acetate ligand exchange also need to be considered [42]. Calculations regarding the detailed reaction mechanism presented elsewhere [20] revealed a lower acetate ligand exchange activation barrier for ${\left\{\mathrm{MnV}\right\}}^{2-}$ compared to ${\left\{\mathrm{MnV}\right\}}^{3-}$ (Figure 3f). Here, this is verified by liquid FT‐IR spectroscopy results, which indicate that for ${\left\{\mathrm{MnV}\right\}}^{3-}$ and ${\left\{\mathrm{MnV}\right\}}^{2-}$, approximately one acetate ligand per cluster is exchanged for water ligands on the timescale of the titration experiments (approximately 60 min; Sections S9 and S10). Further evidence for this ligand exchange is provided by high‐resolution electrospray ionization mass spectrometry (HR‐ESI MS), where increasing amounts of free acetate ligands are observed for ${\left\{\mathrm{MnV}\right\}}^{3-}$ with increasing water content (Sections S11 and 12).

**FIGURE 4:**
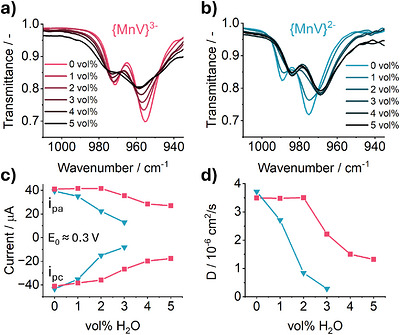
Liquid FT‐IR spectra of (a) ${\left\{\mathrm{MnV}\right\}}^{3-}$ and (b) ${\left\{\mathrm{MnV}\right\}}^{2-}$, showing changes of the characteristic vibrations with variation of the water content. Conditions: concentration 10 mM, path length 0.05 mm. (c) Electrochemical analysis of the anodic (ipa) and cathodic (ipc) peak current changes with varying water contents for ${\left\{\mathrm{MnV}\right\}}^{3-}$ (red) and ${\left\{\mathrm{MnV}\right\}}^{2-}$ (blue). Conditions: concentration 1 mM. (d) Electrochemical analysis of the diffusion coefficients (determined by RS analysis) for ${\left\{\mathrm{MnV}\right\}}^{3-}$ (red) and ${\left\{\mathrm{MnV}\right\}}^{2-}$ (blue). Conditions: concentration 1 mM.

To confirm the initial formation of a hydrogen‐bonded water network around the cluster, we performed a titration study to study the characteristic V═O bands at 930–1000 ${\mathrm{cm}}^{-1}$ using liquid‐phase FT‐IR spectroscopy. Note that under water‐free conditions, we observe the characteristic vibrations at higher wavenumbers for ${\left\{\mathrm{MnV}\right\}}^{2-}$ compared to ${\left\{\mathrm{MnV}\right\}}^{3-}$ (see Figure 4b). This is in line with our previous calculations [41] and arises from changes in electron density at the terminal V═O bonds associated with different charge states. FT‐IR analysis shows that increasing water concentrations leads to a weakening and broadening of the vibrational modes, a feature which is more pronounced for ${\left\{\mathrm{MnV}\right\}}^{3-}$. Here, the two bands virtually merge while for ${\left\{\mathrm{MnV}\right\}}^{2-}$, the characteristic two‐peak pattern is still observed at water contents of 5 vol%. DFT calculations reveal that these changes in peak shape and position can be directly attributed to hydrogen bonding between water molecules of the solvation shell and the V═O groups, which lowers the electron density at the terminal oxo ligands and induces fluctuations in the local electronic structure (Section S13). The formation of a solvation shell is further indicated by 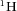 NMR spectroscopy, whereas characteristic decrease in T1 relaxation time of the water protons indicates reduced tumbling rate due to the formation of a hydrogen bonding network with the cluster (Sections S14–S16). In ${\left\{\mathrm{MnV}\right\}}^{2-}$, we also observe the formation of a new species at water contents above 3 vol%. Based on comparison between the solid‐state and liquid FT‐IR spectra, we propose that this effect is due to (cation‐ and solvent‐mediated) aggregation of several clusters [43]. Similar behavior has been reported previously for related polyoxovanadates [44]. Water‐induced cluster aggregation is further supported by electrochemical analyses (Sections S17–S19), which shows less pronounced redox‐events and decreasing diffusion coefficients with increasing water content (Figure 4c,d). In fact, aggregation could also contribute to the disappearance of characteristic cluster‐based 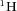, 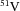 NMR and ESI MS signals as well as the decreasing T1 relaxation times observed (Sections S12 and S15).

#### 2.3.1 Simulation of Multiple ${\left\{\mathrm{MnV}\right\}}^{n-}$

While the calculations so far focused on resolving solvent effects in systems containing a single POM, the experimental observations motivated us to also examine aggregation behavior for the ${\left\{\mathrm{MnV}\right\}}^{n-}$ species. This is relevant since cation/solvent‐mediated supramolecular aggregation is a well‐established phenomenon in POM chemistry [45]. Thus, we set to investigate whether similar aggregation processes occur for ${\left\{\mathrm{MnV}\right\}}^{n-}$ clusters under the conditions relevant to our study. Agglomeration of POMs was thus investigated in the oxidation states ${\left\{\mathrm{MnV}\right\}}^{2-}$ and ${\left\{\mathrm{MnV}\right\}}^{3-}$, each dissolved in 5 vol% water content, as these are the species investigated experimentally (see Section S1 for computational details). Interestingly, visual inspection of the MD trajectories reveals a mild agglomeration tendency for both oxidation states, although more pronounced for ${\left\{\mathrm{MnV}\right\}}^{2-}$.

**FIGURE 5:**
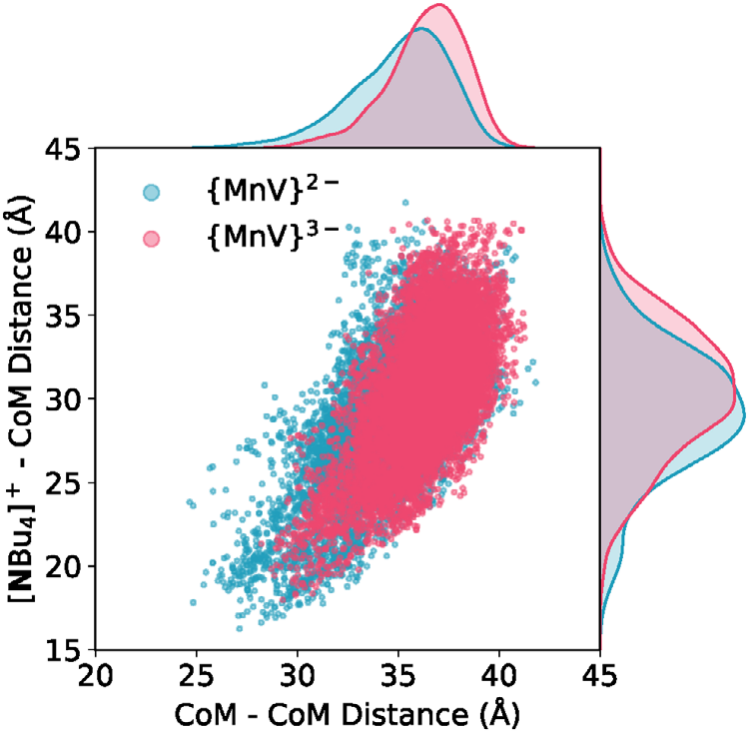
Averaged distance between the counterion ${\left[{\mathrm{NBu}}_{4}\right]}^{+}$ and ${\left\{\mathrm{MnV}\right\}}^{n-}$ versus the averaged pairwise distance between ${\left\{\mathrm{MnV}\right\}}^{n-}$ clusters. Kernel density estimators shown along the axes. Time‐averaged data obtained from 200 ns of two independent systems, each containing five ${\left\{\mathrm{MnV}\right\}}^{3-}$ or five ${\left\{\mathrm{MnV}\right\}}^{2-}$ clusters, respectively, solvated in an acetonitrile/water mixture with 5 vol% water.

In Figure 5, we show the averaged distance between the nitrogen counterion atoms of ${\left[{\mathrm{NBu}}_{4}\right]}^{+}$ and the CoM of ${\left\{\mathrm{MnV}\right\}}^{n-}$, plotted against the pairwise distances between the CoM of individual ${\left\{\mathrm{MnV}\right\}}^{n-}$. Kernel density estimators are shown along the axes, computed according to the procedure of ref. [46]. In the case of ${\left\{\mathrm{MnV}\right\}}^{2-}$, the catalysts get closer to each other compared to ${\left\{\mathrm{MnV}\right\}}^{3-}$. From visual inspection of the simulations, we see that when colloids are formed there are either ${\left[{\mathrm{NBu}}_{4}\right]}^{+}$ ions in between the POMs or none. This is reflected in the correlation between the ${\left[{\mathrm{NBu}}_{4}\right]}^{+}$ to ${\left\{\mathrm{MnV}\right\}}^{n-}$ distance and pairwise ${\left\{\mathrm{MnV}\right\}}^{n-}$ distances. The closer the catalysts get to each other the closer the counterions get to the catalyst. This implies a ${\left[{\mathrm{NBu}}_{4}\right]}^{+}$ mediated agglomeration behavior. This behavior is similar to that found in polyoxovanadates, where tetraethylammonium counterions functioned as a linker to form agglomerates [44].

## 3 Conclusions

This combined computational and experimental study elucidates the solvation environment of a MnV POM WOC across its different oxidation states (${\left\{\mathrm{MnV}\right\}}^{3-}$, ${\left\{\mathrm{MnV}\right\}}^{2-}$, and ${\left\{\mathrm{MnV}\right\}}^{1-}$) in pure water, pure acetonitrile, and acetonitrile/water mixtures. Molecular dynamics simulations reveal pronounced oxidation‐state‐ and charge‐dependent differences in solvation structure, which are confirmed experimentally, highlighting the critical role of microsolvation in governing catalyst stability and reactivity. In mixed solvents, the reduced ${\left\{\mathrm{MnV}\right\}}^{3-}$ species is surrounded by a well‐defined water‐rich solvation shell driven by strong hydrogen bonding and electrostatic interactions, whereas the oxidized ${\left\{\mathrm{MnV}\right\}}^{1-}$ state exhibits a comparatively weaker and homogeneous solvation environment. Three dimensional spatial distribution functions highlight the formation of structured hydration shells around the reduced catalyst, effectively shielding the catalyst from acetonitrile.

Considering that the catalysis requires oxidation of ${\left\{\mathrm{MnV}\right\}}^{3-}$ to ${\left\{\mathrm{MnV}\right\}}^{2-}$, where ligand exchange replaces an acetate ligand with two water molecules, these results have direct implications for the catalytic cycle. The enhanced hydration of the reduced state effectively pre‐organize the catalyst for subsequent water activation, including the nucleophilic water attack. Moreover, the observation that acetonitrile assists in positioning water molecules closer to the catalyst provides a molecular‐level explanation for the experimentally observed enhancement of catalytic performance in solvent mixtures. Overall, this work highlights how the oxidation state of POMs governs their local solvation environment and underscore the importance of explicitly considering solvent effects when interpreting reactivity and stability in molecular water‐oxidation catalysis.

## Author Contributions

S.T. contributed to the force‐field parametrization and validation, performed all molecular dynamics simulations, the data analysis, and prepared the original draft of the manuscript. M.R. performed the synthesis and experimental analyses including spectroscopic measurements as well as electrochemistry. S.M. performed the force‐field parametrization. B.M. supported the electrochemical measurements and evaluated and discussed the data. M.M. set up the NMR experiments and analyzed and discussed the NMR spectroscopy data. C.S. was responsible for experimental conceptualization, acquisition of funding and experimental supervision. L.G. was responsible for project conceptualization, acquisition of funding, theoretical planning and supervision as well as writing and editing of the manuscript. All authors contributed to the review, editing, and final approval of the manuscript.

## Conflicts of Interest

The authors declare no conflict of interest.

## Supporting information


**Supporting File 1**: All data supporting the findings of this study are provided in the main text and/or the Supporting Information.

**Supporting File 2**: anie72128‐sup‐0002‐Data.zip.

## Data Availability

The data that support the findings of this study are available at zenodo.org under identifier https://doi.org/10.5281/zenodo.18037298 (identifier number 18037298).
