# Supplementary material for: Oxidation State Determines Solvent Structure Around a Manganese–Vanadium Polyoxometalate Water‐Oxidation Catalyst
Source: Angew Chem Int Ed Engl. 2026 Apr 16;65(24):e9168848. doi: 10.1002/anie.9168848 (PMC13245616; doi:10.1002/anie.9168848)
Supplement: Supplementary file 1 — Supporting File 1: All data supporting the findings of this study are provided in the main text and/or the Supporting Information. [file ANIE-65-e9168848-s001.pdf]

Supporting Information for:

Oxidation State Determines Solvent Structure Around a Manganese-Vanadium Polyoxometalate Water-Oxidation Catalyst

Simon Tippner [1,2], Moritz Remmers [3], Sebastian Mai [1], Boris Mashtakov [3], Mihail Mondeshki [3], Carsten Streb [3], Leticia González [1]

[1] Institute of Theoretical Chemistry, Faculty of Chemistry, University of Vienna, Währinger Str. 17, 1090 Vienna, Austria.

[2] University of Vienna, Vienna Doctoral School in Chemistry (DoSChem), Währinger Str. 42, 1090 Vienna, Austria.

[3] Department of Chemistry, Johannes Gutenberg University Mainz Duesbergweg 10–14, 55128 Mainz, Germany.

Contents

S1 Molecular Dynamics Simulations S3

S1.1 Force Fields and Electronic Structure S5

S2 Densities of Acetonitrile/Water Mixtures S17

S3 Radial distribution functions between solvents and individual atoms in {MnV}$^{n-}$ in pure solvents S19

S4 Radial distribution functions between center of mass of {MnV}*n*- and solvent molecules (water and acetonitrile) S21

S5 Radial distribution functions between solvents and individual atoms in {MnV}$^{n-}$ in acetonitrile/water mixtures (5 vol% water) S22

S6 Hydrogen bonds in acetonitrile/water mixtures (5 vol% water) S24

S7 Instrumentation S25

S8 UV-VIS Spectroscopy S27

S9 FTIR Spectroscopy S28

S10 Liquid IR Spectroscopy: Acetic Acid S29

S11 ESI Mass Spectroscopy: {MnV}$^{3-}$ and {MnV}$^{2-}$ S31

S12 ESI Mass Spectroscopy: Titration of H_2O S34

S13 Computed IR Spectra S35

S14 NMR Spectroscopy of {MnV}$^{3-}$: Peak Assignment **S36**

S15 NMR Spectroscopy: Titration with H_2O **S37**

S16 ^{1}H-NMR Spectroscopy: T1 Measurements **S38**

S17 Electrochemistry: Electrochemical Generation of {MnV}$^{2-}$ **S39**

S18 Electrochemistry: Titration of H_2O **S40**

S19 Randles–Ševčík Analysis: Titration of H_2O **S41**

S1 Molecular Dynamics Simulations

We performed three sets of molecular dynamics (MD) simulations: (i) solvent system with no catalyst present; (ii) solvent systems with one $\{MnV\}^{n-}$ catalyst present; and (iii) solvent system of 5 vol% water including 20 $\{MnV\}^{n-}$ catalysts. All boxes were constructed with PACKMOL[1] using a box size of $(70\ \text{Å})^3$ containing the appropriate number of water, acetonitrile, POMs and counterions, as listed in Table S1.

Table S1: Number of molecules used for MD simulations. The systems are grouped into (i) solvent-only boxes, (ii) systems containing one POM catalyst, and (iii) systems containing 20 POM catalysts. Tetrabutylammonium counterions are denoted as TBA^+.

**(i) Solvent-only systems**					
Box Size	$x(CH_3CN)$	# CH_3CN	# H_2O	Counterion	Catalyst
70^3 Å^3	0.0	0	11466	-	-
70^3 Å^3	0.25	2056	6168	-	-
70^3 Å^3	0.5	3123	3123	-	-
70^3 Å^3	0.75	3685	1228	-	-
70^3 Å^3	1.0	3955	0	-	-
**(ii) Systems containing one POM**					
Box Size	vol% H_2O	# CH_3CN	# H_2O	Counterion	Catalyst
70^3 Å^3	0.0	4978	-	3 TBA^+	Mn3344
70^3 Å^3	0.5	3906	57	3 TBA^+	Mn3344
70^3 Å^3	1.0	3896	114	3 TBA^+	Mn3344
70^3 Å^3	2.0	3875	229	3 TBA^+	Mn3344
70^3 Å^3	3.0	3852	334	3 TBA^+	Mn3344
70^3 Å^3	4.0	3828	461	3 TBA^+	Mn3344
70^3 Å^3	5.0	3802	578	3 TBA^+	Mn3344
70^3 Å^3	6.0	3776	697	3 TBA^+	Mn3344
70^3 Å^3	8.0	3719	935	3 TBA^+	Mn3344
70^3 Å^3	10.0	3659	1175	3 TBA^+	Mn3344
70^3 Å^3	100.0	-	11343	3 TBA^+	Mn3344
70^3 Å^3	0.0	3922	-	2 TBA^+	Mn3444
70^3 Å^3	0.5	3912	57	2 TBA^+	Mn3444
70^3 Å^3	1.0	3902	114	2 TBA^+	Mn3444
70^3 Å^3	2.0	3881	229	2 TBA^+	Mn3444
70^3 Å^3	3.0	3858	345	2 TBA^+	Mn3444
70^3 Å^3	4.0	3834	462	2 TBA^+	Mn3444
70^3 Å^3	5.0	3808	579	2 TBA^+	Mn3444
70^3 Å^3	6.0	3781	698	2 TBA^+	Mn3444
70^3 Å^3	8.0	3724	936	2 TBA^+	Mn3444
70^3 Å^3	10.0	3664	1177	2 TBA^+	Mn3444
70^3 Å^3	100.0	-	11356	2 TBA^+	Mn3444
70^3 Å^3	0.0	3928	-	1 TBA^+	Mn4444
70^3 Å^3	0.5	3918	57	1 TBA^+	Mn4444
70^3 Å^3	1.0	3908	114	1 TBA^+	Mn4444
70^3 Å^3	2.0	3886	229	1 TBA^+	Mn4444
70^3 Å^3	3.0	3863	345	1 TBA^+	Mn4444
70^3 Å^3	4.0	3839	462	1 TBA^+	Mn4444
70^3 Å^3	5.0	3814	580	1 TBA^+	Mn4444
70^3 Å^3	6.0	3787	699	1 TBA^+	Mn4444
70^3 Å^3	8.0	3730	937	1 TBA^+	Mn4444
70^3 Å^3	10.0	3669	1178	1 TBA^+	Mn4444
70^3 Å^3	100.0	-	11370	1 TBA^+	Mn4444
**(iii) Systems containing 20 POM catalysts**					
Box Size	vol% H_2O	# CH_3CN	# H_2O	Counterion	Catalyst
70^3 Å^3	5	3109	473	15 TBA^+	5 × Mn3344
70^3 Å^3	5	3112	478	10 TBA^+	5 × Mn3444

S1.1 Force Fields and Electronic Structure

Partial atomic charges for acetonitrile and TBA^+ were obtained based on the restrained electrostatic potential method RESP,[2] using `antechamber`[3,4], derived from quantum mechanical (QM) calculations from geometry optimizations at B3LYP/ def2-SVP and HF/def2-SVP level of theory.[5–8] Dispersion was treated with Grimme's D3(BJ) correction.[9,10] The QM calculations were carried out using Gaussian 16.[11] For acetonitrile, in addition to the RESP charges derived from quantum mechanical calculations, we also employed the six-site model developed by Nikitin *et al.*[12].

The charges are summarized in Table S2. Figure S1 shows the three-dimensional structure of CH_3CN and TBA^+, including the atom labels.

Table S2: Atomic partial charges used for acetonitrile (CH_3CN) and tetrabutylammonium (TBA^+).

Species	Atom	Charge q	Species	Atom	Charge q
**Acetonitrile (CH_3CN)**					
CH_3CN	C1	-0.550300	CH_3CN	H2	0.190400
CH_3CN	C2	0.491700	CH_3CN	H3	0.190400
CH_3CN	H1	0.190400	CH_3CN	N	-0.512600
**Tetrabutylammonium (TBA^+)**					
TBA^+	C1	-0.1783	TBA^+	H1	0.0750
TBA^+	C2	-0.0231	TBA^+	H2	0.0750
TBA^+	C3	0.1457	TBA^+	H3	0.0232
TBA^+	C4	-0.2503	TBA^+	H4	0.0232
TBA^+	C5	-0.1783	TBA^+	H5	0.0013
TBA^+	C6	-0.0231	TBA^+	H6	0.0013
TBA^+	C7	0.1457	TBA^+	H7	0.0737
TBA^+	C8	-0.2503	TBA^+	H8	0.0737
TBA^+	C9	-0.1783	TBA^+	H9	0.0737
TBA^+	C10	-0.0231	TBA^+	H10	0.0750
TBA^+	C11	0.1457	TBA^+	H11	0.0750
TBA^+	C12	-0.2503	TBA^+	H12	0.0232
TBA^+	C13	-0.1783	TBA^+	H13	0.0232
TBA^+	C14	-0.0231	TBA^+	H14	0.0013
TBA^+	C15	0.1457	TBA^+	H15	0.0013
TBA^+	C16	-0.2503	TBA^+	H16	0.0737
TBA^+	N1	0.5433	TBA^+	H17	0.0737
TBA^+			TBA^+	H18	0.0737
TBA^+			TBA^+	H19	0.0750
TBA^+			TBA^+	H20	0.0750
TBA^+			TBA^+	H21	0.0232
TBA^+			TBA^+	H22	0.0232
TBA^+			TBA^+	H23	0.0013
TBA^+			TBA^+	H24	0.0013
TBA^+			TBA^+	H25	0.0737
TBA^+			TBA^+	H26	0.0737
TBA^+			TBA^+	H27	0.0737
TBA^+			TBA^+	H28	0.0750
TBA^+			TBA^+	H29	0.0750
TBA^+			TBA^+	H30	0.0232
TBA^+			TBA^+	H31	0.0232
TBA^+			TBA^+	H32	0.0013
TBA^+			TBA^+	H33	0.0013
TBA^+			TBA^+	H34	0.0737
TBA^+			TBA^+	H35	0.0737
TBA^+			TBA^+	H36	0.0737

Water was modeled with TIP3P,[13] SPC/Fw,[14] and q-SPC/Fw.[15]

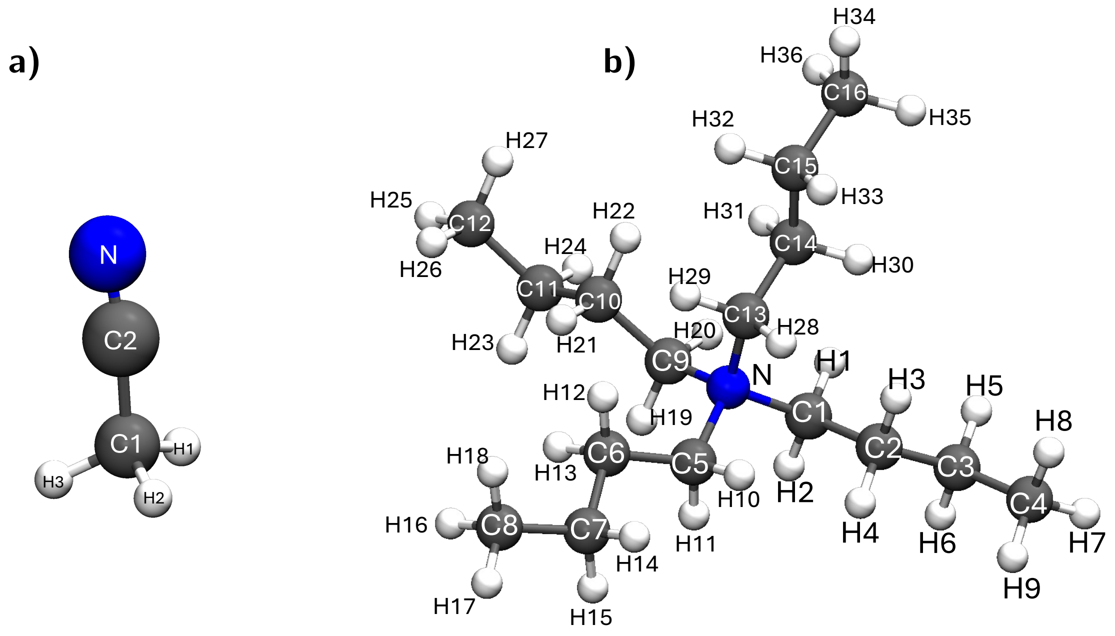

Figure S1: Three-dimensional structure of a) CH_3CN and TBA^+.

All simulations were performed with AMBER 22[?] and accelerated by using its GPU (CUDA) version `pmemd.cuda`[16,17]. A cutoff of 10 Å was applied for non-bonded interaction terms. For TIP3P water, we used a 2 fs time step with SHAKE[18] on water bonds/angles. For flexible water models (SPC/Fw, q-SPC/Fw), SHAKE was disabled and a 0.1 fs time step was used. Each system was minimized for 10,000 steps (5,000 steepest descent + 5,000 conjugate gradient), heated to 100 K over 26.25 ps, then to 300 K over 25 ps under periodic NVT with a Langevin thermostat. Subsequently, NPT (isotropic pressure coupling) equilibration was run for 1 ns, followed by production runs of 10 ns for pure solvent benchmarks and 100 ns for POM-containing systems. Radial distribution functions (RDFs) and three-dimensional spatial distribution functions, which map the average number of atoms within specific volume elements per frame were computed with `CPPTRAJ` (AmberTools).[19] The volume elements are cubic voxels with an edge length of 0.5 Å. The average number of solvent atoms per voxel is obtained from the voxel volume and the molar density of the solvent.[20]

The POM in its oxidation states $\{MnV\}^{3-}$ (also denoted as Mn3344), $\{MnV\}^{2-}$ (Mn3444), and $\{MnV\}^{1-}$ (Mn4444) was described by a force field revised from Ref. 21. Atom type definitions and some angle parameters were directly taken from that reference. The bond stretch parameters, the remaining angle parameters, and the dihedral parameters were revised to obey symmetry and reproduce the bond length distributions of a quantum mechanics/molecular mechanics reference trajectory. These reference trajectories were computed as described in Ref. 21, using BP86/def2-SVP and LANL2DZ in Terachem to propagate $\{MnV\}^{3-}$ and Na^+ counterions in a 58 Å box of acetonitrile over 5 ps. All dihedral parameters involving two Mn atoms were set to zero, either because they are redundant with angle parameters inside the cage or because they are ill-defined dihedrals involving two collinear bonds. Dihedral potentials involving V and C atoms were kept to avoid twisting of the V–O–V bridges and deplanarization of the acetates. Partial charges were revised with the CHELPG procedure implemented in ORCA,[?] using D3-BP86/ZORA-def2-SVP (ZORA-def2-TZVP for Mn, V, O) without CPCM.

All force field parameters can be found in the force_field.zip file. In the following, we summarize the equations for the potential energy terms to put the parameters into context. Furthermore, all force-field parameters are provided in Table S3-S11.

$$E_{\text{bonds}} = \sum_{i}^{n_{\text{bonds}}} K_{r,i}(r_i - r_{i,\text{eq}})^2 \tag{1}$$

$$E_{\text{angles}} = \sum_{i}^{n_{\text{angles}}} K_{\alpha,i}(\alpha_i - \alpha_{i,\text{eq}})^2 \tag{2}$$

$$E_{\text{dihedrals}} = \sum_{i}^{n_{\text{dihedrals}}} V_i\,(1 + \cos(n_i\phi_i - \gamma_i)) \tag{3}$$

$$E_{\text{LJ}} = \sum_{i<j}^{n_{\text{atoms}}} \sqrt{\varepsilon_i\varepsilon_j}\left[\left(\frac{r_{i,\text{min}} + r_{i,\text{min}}}{2r_{ij}}\right)^{12} - 2\left(\frac{r_{i,\text{min}} + r_{i,\text{min}}}{2r_{ij}}\right)^{6}\right] \tag{4}$$

$$E_{\text{Coulomb}} = \sum_{i<j}^{n_{\text{atoms}}} \frac{q_i q_j}{4\pi\varepsilon_0 r_{ij}} \tag{5}$$

with $K_{r,i}$ in kcal mol^{-1}Å^{-2} and $r_{i,\text{eq}}$ in Å, $K_{\alpha,i}$ in kcal mol^{-1}rad^{-2} and $\alpha_{i,\text{eq}}$ in degree, V_i (half the torsion barrier) in kcal mol^{-1}, n_i the periodicity, and γ_i the phase in degrees, ε_i, ε_j in kcal mol^{-1} and $r_{i,\text{min}}$, $r_{j,\text{min}}$ in Å; q_i, q_j are given in multiples of the elementary charge and $\frac{1}{4\pi\varepsilon_0} = 332.05$ Å kcal mol^{-1}.

Table S3: List of all obtained parameters for **bonds**. Parameters $K_{r,i}$ are in kcal mol^{-1}Å^{-2} and $r_{i,\mathrm{eq}}$ in Å.

		— [Mn_4^{IV}] —		— [$Mn^{III}Mn_3^{IV}$] —		— [$Mn_2^{III}Mn_2^{IV}$] —	
Atom 1	Atom 2	$K_{r,i}$	$r_{i,\mathrm{eq}}$	$K_{r,i}$	$r_{i,\mathrm{eq}}$	$K_{r,i}$	$r_{i,\mathrm{eq}}$
MA	o	260.00	2.0527	260.00	2.1097	180.00	2.4153
MA	OE	400.00	1.9027	400.00	1.9239	400.00	1.9587
MA	OF	400.00	1.9049	400.00	1.9409	400.00	1.9691
MA	OJ	160.00	2.0830	160.00	2.0070	100.00	2.3663
MA	OK	300.00	2.0343	300.00	2.0308	300.00	2.0670
MA	OL	300.00	2.0313	300.00	2.0594	300.00	2.0896
MB	OK	500.00	1.9199	500.00	1.9401	500.00	1.9085
MB	OL	500.00	1.9265	500.00	1.9073	500.00	1.8920
MB	OM	500.00	1.9265	500.00	1.9073	500.00	1.8920
MB	ON	350.00	2.0447	350.00	2.0858	350.00	2.0763
MB	OO	350.00	2.0463	350.00	2.0137	350.00	2.0689
MB	OP	350.00	2.0447	350.00	2.0858	350.00	2.0763
MC	o	260.00	2.0640	180.00	2.3514	260.00	2.2179
MC	OD	400.00	1.8978	400.00	1.9432	400.00	1.9453
MC	OI	400.00	1.8978	400.00	1.9432	400.00	1.9453
MC	OJ	160.00	2.0909	100.00	2.4833	160.00	1.9332
MC	OL	300.00	2.0408	300.00	2.0913	300.00	2.0693
MC	OM	300.00	2.0408	300.00	2.0913	300.00	2.0693
MD	o	260.00	2.0527	260.00	2.1097	180.00	2.4153
MD	OG	400.00	1.9049	400.00	1.9409	400.00	1.9691
MD	OH	400.00	1.9027	400.00	1.9239	400.00	1.9587
MD	OJ	160.00	2.0830	160.00	2.0070	100.00	2.3663
MD	OK	300.00	2.0343	300.00	2.0308	300.00	2.0670
MD	OM	300.00	2.0313	300.00	2.0594	300.00	2.0896
VA	OA	1000.00	1.6047	1000.00	1.6168	1000.00	1.6291
VA	OB	600.00	1.8108	600.00	1.8059	600.00	1.8103
VA	OR	600.00	1.8108	600.00	1.8117	600.00	1.8048
VB	OB	600.00	1.8362	600.00	1.8491	600.00	1.8637
VB	OC	1000.00	1.6078	1000.00	1.6210	1000.00	1.6324
VB	OD	600.00	1.8019	600.00	1.7822	600.00	1.8034
VB	OE	600.00	1.8019	600.00	1.8015	600.00	1.7863
VB	OH	600.00	1.8019	600.00	1.8015	600.00	1.7863
VB	OI	600.00	1.8019	600.00	1.7822	600.00	1.8034
VC	OF	600.00	1.8018	600.00	1.8044	600.00	1.7819
VC	OG	600.00	1.8018	600.00	1.8044	600.00	1.7819
VC	OQ	1000.00	1.6079	1000.00	1.6180	1000.00	1.6368
VC	OR	600.00	1.8362	600.00	1.8524	600.00	1.8582
c	o	1070.00	1.2982	1070.00	1.2869	1070.00	1.2772
c	ON	800.00	1.3072	800.00	1.3043	800.00	1.3131
c	OO	800.00	1.3073	800.00	1.3190	800.00	1.3038
c	OP	800.00	1.3072	800.00	1.3043	800.00	1.3131

Table S4: List of all obtained parameters for **angles**. Parameters $K_{\alpha,i}$ are in kcal mol^{-1}rad^{-2} and $\alpha_{i,\mathrm{eq}}$ in degree.

			— [Mn_4^{IV}] —		— [$Mn^{III}Mn_3^{IV}$] —		— [$Mn_2^{III}Mn_2^{IV}$] —					— [Mn_4^{IV}] —		— [$Mn^{III}Mn_3^{IV}$] —		— [$Mn_2^{III}Mn_2^{IV}$] —	
Atom 1	Atom 2	Atom 3	$K_{\alpha,i}$	$\alpha_{i,\mathrm{eq}}$	$K_{\alpha,i}$	$\alpha_{i,\mathrm{eq}}$	$K_{\alpha,i}$	$\alpha_{i,\mathrm{eq}}$	Atom 1	Atom 2	Atom 3	$K_{\alpha,i}$	$\alpha_{i,\mathrm{eq}}$	$K_{\alpha,i}$	$\alpha_{i,\mathrm{eq}}$	$K_{\alpha,i}$	$\alpha_{i,\mathrm{eq}}$
MA	OJ	MC	600	97.592	600	94.095	600	96.816	OG	MD	o	200	96.489	200	95.087	200	102.759
MA	OJ	MD	600	97.936	600	100.816	600	87.837	OH	MD	o	200	96.929	200	95.758	200	100.726
MC	OJ	MD	600	97.592	600	94.095	600	96.816	OG	MD	OH	200	95.482	200	95.960	200	96.535
MA	OK	MB	600	96.610	600	96.236	600	96.530	OJ	MD	o	200	164.242	200	166.372	200	157.259
MA	OK	MD	600	99.949	600	97.752	600	103.722	OJ	MD	OG	200	93.748	200	91.567	200	96.310
MB	OK	MD	600	96.610	600	96.236	600	96.530	OJ	MD	OH	200	93.481	200	94.944	200	87.990
MA	OL	MB	600	96.588	600	96.100	600	96.543	OK	MD	o	200	86.749	200	86.688	200	84.383
MA	OL	MC	600	99.645	600	104.118	600	101.086	OK	MD	OG	150	91.177	150	91.836	150	91.427
MB	OL	MC	600	96.771	600	97.684	600	96.117	OK	MD	OH	150	171.140	150	170.897	150	168.690
MB	OM	MC	600	96.771	600	97.684	600	96.117	OK	MD	OJ	150	80.541	150	80.588	150	82.936
MB	OM	MD	600	96.588	600	96.100	600	96.543	OK	MD	OM	200	80.411	200	80.565	200	78.996
MC	OM	MD	600	99.645	600	104.118	600	101.086	OM	MD	o	200	86.516	200	85.139	200	83.023
OE	MA	o	200	96.929	200	95.758	200	100.726	OM	MD	OG	150	171.275	150	172.802	150	168.592
OF	MA	o	200	96.489	200	95.087	200	102.759	OM	MD	OH	150	91.532	150	90.532	150	91.122
OE	MA	OF	200	95.482	200	95.960	200	96.535	OM	MD	OJ	150	80.858	150	85.918	150	75.308
OJ	MA	o	200	164.242	200	166.372	200	157.259	MC	OD	VB	200	122.000	200	122.000	200	122.000
OJ	MA	OE	200	93.481	200	94.944	200	87.990	MA	OE	VB	200	122.000	200	122.000	200	122.000
OJ	MA	OF	200	93.748	200	91.567	200	96.310	MA	OF	VC	200	127.000	200	127.000	200	127.000
OK	MA	o	200	86.749	200	86.688	200	84.383	MD	OG	VC	200	127.000	200	127.000	200	127.000
OK	MA	OE	150	171.140	150	170.897	150	168.690	MD	OH	VB	200	122.000	200	122.000	200	122.000
OK	MA	OF	150	91.177	150	91.836	150	91.427	MC	OI	VB	200	122.000	200	122.000	200	122.000
OK	MA	OJ	150	80.541	150	80.588	150	82.936	VA	OB	VB	100	142.428	100	142.249	100	137.918
OK	MA	OL	200	80.411	200	80.565	200	78.996	VA	OR	VC	100	142.366	100	140.035	100	145.147
OL	MA	o	200	86.516	200	85.139	200	83.023	OA	VA	OB	160	107.752	160	107.350	160	106.190
OL	MA	OE	150	91.532	150	90.532	150	91.122	OB	VA	OB	140	111.152	140	110.433	140	113.446
OL	MA	OF	150	171.275	150	172.802	150	168.592	OA	VA	OR	160	107.781	160	107.207	160	106.354
OL	MA	OJ	150	80.858	150	85.918	150	75.308	OB	VA	OR	140	111.006	140	112.057	140	112.075
OK	MB	OL	600	85.599	600	86.532	600	86.951	OB	VB	OC	160	110.644	160	109.758	160	106.368
OK	MB	OM	600	85.599	600	86.532	600	86.951	OB	VB	OD	140	109.940	140	110.210	140	111.061
OK	MB	ON	200	88.406	200	87.751	200	92.916	OB	VB	OE	140	110.058	140	108.102	140	111.534
OK	MB	OO	200	171.536	200	176.716	200	174.898	OR	VC	OF	140	110.531	140	112.555	140	108.838
OK	MB	OP	200	88.406	200	87.751	200	92.916	OR	VC	OG	140	110.531	140	112.555	140	108.838
OL	MB	OM	600	85.497	600	85.265	600	87.475	OB	VB	OH	140	110.058	140	108.102	140	111.534
OL	MB	ON	200	172.243	200	172.834	200	177.461	OB	VB	OI	140	109.940	140	110.210	140	111.061
OL	MB	OO	200	87.781	200	92.867	200	89.435	OC	VB	OD	160	109.747	160	109.735	160	106.943
OL	MB	OP	200	88.287	200	89.277	200	91.829	OC	VB	OE	160	109.813	160	109.443	160	107.711
OM	MB	ON	200	88.287	200	89.277	200	91.829	OQ	VC	OF	160	109.080	160	106.563	160	109.374
OM	MB	OO	200	87.781	200	92.867	200	89.435	OQ	VC	OG	160	109.080	160	106.563	160	109.374
OM	MB	OP	200	172.243	200	172.834	200	177.461	OC	VB	OH	160	109.813	160	109.443	160	107.711
ON	MB	OO	150	96.673	150	91.850	150	89.674	OC	VB	OI	160	109.747	160	109.735	160	106.943
ON	MB	OP	150	96.480	150	94.775	150	87.829	OD	VB	OE	140	106.317	140	109.337	140	112.608
OO	MB	OP	150	96.673	150	91.850	150	89.674	OF	VC	OG	140	107.482	140	111.477	140	110.904
OD	MC	o	200	96.702	200	104.786	200	94.230	OH	VB	OI	140	106.317	140	109.337	140	112.608
OI	MC	o	200	96.702	200	104.786	200	94.230	OQ	VC	OR	160	109.839	160	106.318	160	109.289
OD	MC	OI	200	95.413	200	96.472	200	97.786	MA	o	c	250	124.500	250	124.500	250	124.500
OJ	MC	o	200	164.301	200	154.280	200	169.481	MC	o	c	250	125.800	250	125.800	250	125.800
OJ	MC	OD	200	93.543	200	91.852	200	92.404	MD	o	c	250	124.500	250	124.500	250	124.500
OJ	MC	OI	200	93.543	200	91.852	200	92.404	MB	ON	c	250	124.500	250	124.500	250	124.500
OL	MC	o	200	86.644	200	85.221	200	84.746	MB	OO	c	250	122.000	250	122.000	250	122.000
OL	MC	OD	150	91.494	150	91.334	150	90.694	MB	OP	c	250	124.500	250	124.500	250	124.500
OL	MC	OI	150	171.190	150	164.744	150	170.774	c3	c	o	84.6	118.400	84.6	118.400	84.6	118.400
OL	MC	OJ	150	80.734	150	74.217	150	86.521	c3	c	ON	84.6	118.000	84.6	118.000	84.6	118.000
OL	MC	OM	200	80.234	200	77.378	200	79.761	c3	c	OO	84.6	118.000	84.6	118.000	84.6	118.000
OM	MC	o	200	86.644	200	85.221	200	84.746	c3	c	OP	84.6	118.000	84.6	118.000	84.6	118.000
OM	MC	OD	150	171.190	150	164.744	150	170.774	OP	c	o	118.8	123.600	118.8	123.600	118.8	123.600
OM	MC	OI	150	91.494	150	91.334	150	90.694	ON	c	o	118.8	123.600	118.8	123.600	118.8	123.600
OM	MC	OJ	150	80.734	150	74.217	150	86.521	OO	c	o	118.8	123.600	118.8	123.600	118.8	123.600

Table S5: List of all obtained parameters for **dihedrals**. Parameters V_i are in kcal mol^{-1}, γ_i is in degrees, and n_i is unitless.

				— $[Mn^{IV}_4]$ —			— $[Mn^{III}Mn^{IV}_3]$ —			— $[Mn^{III}_2Mn^{IV}_2]$ —		
Atom 1	Atom 2	Atom 3	Atom 4	V_i	γ_i	n_i	V_i	γ_i	n_i	V_i	γ_i	n_i
MA	OJ	MC	OL	0.00	180.0	1	0.00	180.0	1	0.00	180.0	1
MA	OJ	MC	OM	0.00	180.0	1	0.00	180.0	1	0.00	180.0	1
MA	OJ	MD	OK	0.00	180.0	1	0.00	180.0	1	0.00	180.0	1
MA	OJ	MD	OM	0.00	180.0	1	0.00	180.0	1	0.00	180.0	1
MA	OK	MB	OL	0.00	180.0	1	0.00	180.0	1	0.00	180.0	1
MA	OK	MB	OM	0.00	180.0	1	0.00	180.0	1	0.00	180.0	1
MA	OK	MD	OJ	0.00	180.0	1	0.00	180.0	1	0.00	180.0	1
MA	OK	MD	OM	0.00	180.0	1	0.00	180.0	1	0.00	180.0	1
MA	OL	MB	OK	0.00	180.0	1	0.00	180.0	1	0.00	180.0	1
MA	OL	MB	OM	0.00	180.0	1	0.00	180.0	1	0.00	180.0	1
MA	OL	MC	OJ	0.00	180.0	1	0.00	180.0	1	0.00	180.0	1
MA	OL	MC	OM	0.00	180.0	1	0.00	180.0	1	0.00	180.0	1
MB	OK	MA	OJ	0.00	180.0	1	0.00	180.0	1	0.00	180.0	1
MB	OK	MA	OL	0.00	180.0	1	0.00	180.0	1	0.00	180.0	1
MB	OK	MD	OJ	0.00	180.0	1	0.00	180.0	1	0.00	180.0	1
MB	OK	MD	OM	0.00	180.0	1	0.00	180.0	1	0.00	180.0	1
MB	OL	MA	OJ	0.00	180.0	1	0.00	180.0	1	0.00	180.0	1
MB	OL	MA	OK	0.00	180.0	1	0.00	180.0	1	0.00	180.0	1
MB	OL	MC	OJ	0.00	180.0	1	0.00	180.0	1	0.00	180.0	1
MB	OL	MC	OM	0.00	180.0	1	0.00	180.0	1	0.00	180.0	1
MB	OM	MC	OJ	0.00	180.0	1	0.00	180.0	1	0.00	180.0	1
MB	OM	MC	OL	0.00	180.0	1	0.00	180.0	1	0.00	180.0	1
MB	OM	MD	OJ	0.00	180.0	1	0.00	180.0	1	0.00	180.0	1
MB	OM	MD	OK	0.00	180.0	1	0.00	180.0	1	0.00	180.0	1
MC	OJ	MA	OK	0.00	180.0	1	0.00	180.0	1	0.00	180.0	1
MC	OJ	MA	OL	0.00	180.0	1	0.00	180.0	1	0.00	180.0	1
MC	OJ	MD	OK	0.00	180.0	1	0.00	180.0	1	0.00	180.0	1
MC	OJ	MD	OM	0.00	180.0	1	0.00	180.0	1	0.00	180.0	1
MC	OL	MA	OJ	0.00	180.0	1	0.00	180.0	1	0.00	180.0	1
MC	OL	MA	OK	0.00	180.0	1	0.00	180.0	1	0.00	180.0	1
MC	OL	MB	OK	0.00	180.0	1	0.00	180.0	1	0.00	180.0	1
MC	OL	MB	OM	0.00	180.0	1	0.00	180.0	1	0.00	180.0	1
MC	OM	MB	OK	0.00	180.0	1	0.00	180.0	1	0.00	180.0	1
MC	OM	MB	OL	0.00	180.0	1	0.00	180.0	1	0.00	180.0	1
MC	OM	MD	OJ	0.00	180.0	1	0.00	180.0	1	0.00	180.0	1
MC	OM	MD	OK	0.00	180.0	1	0.00	180.0	1	0.00	180.0	1

Table S6: Continuation of Table S5.

				— $[Mn^{IV}_4]$ —			— $[Mn^{III}Mn^{IV}_3]$ —			— $[Mn^{III}_2Mn^{IV}_2]$ —		
Atom 1	Atom 2	Atom 3	Atom 4	V_i	γ_i	n_i	V_i	γ_i	n_i	V_i	γ_i	n_i
MD	OJ	MA	OK	0.00	180.0	1	0.00	180.0	1	0.00	180.0	1
MD	OJ	MA	OL	0.00	180.0	1	0.00	180.0	1	0.00	180.0	1
MD	OJ	MC	OL	0.00	180.0	1	0.00	180.0	1	0.00	180.0	1
MD	OJ	MC	OM	0.00	180.0	1	0.00	180.0	1	0.00	180.0	1
MD	OK	MA	OJ	0.00	180.0	1	0.00	180.0	1	0.00	180.0	1
MD	OK	MA	OL	0.00	180.0	1	0.00	180.0	1	0.00	180.0	1
MD	OK	MB	OL	0.00	180.0	1	0.00	180.0	1	0.00	180.0	1
MD	OK	MB	OM	0.00	180.0	1	0.00	180.0	1	0.00	180.0	1
MD	OM	MB	OK	0.00	180.0	1	0.00	180.0	1	0.00	180.0	1
MD	OM	MB	OL	0.00	180.0	1	0.00	180.0	1	0.00	180.0	1
MD	OM	MC	OJ	0.00	180.0	1	0.00	180.0	1	0.00	180.0	1
MD	OM	MC	OL	0.00	180.0	1	0.00	180.0	1	0.00	180.0	1
MA	OJ	MC	o	0.00	0.0	1	0.00	0.0	1	0.00	0.0	1
MA	OJ	MC	OD	0.00	180.0	1	0.00	180.0	1	0.00	180.0	1
MA	OJ	MC	OI	0.00	180.0	1	0.00	180.0	1	0.00	180.0	1
MA	OJ	MD	o	0.00	0.0	1	0.00	0.0	1	0.00	0.0	1
MA	OJ	MD	OG	0.00	180.0	1	0.00	180.0	1	0.00	180.0	1
MA	OJ	MD	OH	0.00	180.0	1	0.00	180.0	1	0.00	180.0	1
MA	OK	MB	ON	0.00	180.0	1	0.00	180.0	1	0.00	180.0	1
MA	OK	MB	OO	0.00	0.0	1	0.00	0.0	1	0.00	0.0	1
MA	OK	MB	OP	0.00	180.0	1	0.00	180.0	1	0.00	180.0	1
MA	OK	MD	o	0.00	180.0	1	0.00	180.0	1	0.00	180.0	1
MA	OK	MD	OG	0.00	180.0	1	0.00	180.0	1	0.00	180.0	1
MA	OK	MD	OH	0.00	0.0	1	0.00	0.0	1	0.00	0.0	1
MA	OL	MB	ON	0.00	0.0	1	0.00	0.0	1	0.00	0.0	1
MA	OL	MB	OO	0.00	180.0	1	0.00	180.0	1	0.00	180.0	1
MA	OL	MB	OP	0.00	180.0	1	0.00	180.0	1	0.00	180.0	1
MA	OL	MC	o	0.00	180.0	1	0.00	180.0	1	0.00	180.0	1
MA	OL	MC	OD	0.00	180.0	1	0.00	180.0	1	0.00	180.0	1
MA	OL	MC	OI	0.00	0.0	1	0.00	0.0	1	0.00	0.0	1
MB	OK	MA	o	0.00	180.0	1	0.00	180.0	1	0.00	180.0	1
MB	OK	MA	OE	0.00	0.0	1	0.00	0.0	1	0.00	0.0	1
MB	OK	MA	OF	0.00	180.0	1	0.00	180.0	1	0.00	180.0	1
MB	OK	MD	o	0.00	180.0	1	0.00	180.0	1	0.00	180.0	1
MB	OK	MD	OG	0.00	180.0	1	0.00	180.0	1	0.00	180.0	1
MB	OK	MD	OH	0.00	0.0	1	0.00	0.0	1	0.00	0.0	1
MB	OL	MA	o	0.00	180.0	1	0.00	180.0	1	0.00	180.0	1
MB	OL	MA	OE	0.00	180.0	1	0.00	180.0	1	0.00	180.0	1
MB	OL	MA	OF	0.00	0.0	1	0.00	0.0	1	0.00	0.0	1
MB	OL	MC	o	0.00	180.0	1	0.00	180.0	1	0.00	180.0	1
MB	OL	MC	OD	0.00	180.0	1	0.00	180.0	1	0.00	180.0	1
MB	OL	MC	OI	0.00	0.0	1	0.00	0.0	1	0.00	0.0	1
MB	OM	MC	o	0.00	180.0	1	0.00	180.0	1	0.00	180.0	1
MB	OM	MC	OD	0.00	0.0	1	0.00	0.0	1	0.00	0.0	1
MB	OM	MC	OI	0.00	180.0	1	0.00	180.0	1	0.00	180.0	1
MB	OM	MD	o	0.00	180.0	1	0.00	180.0	1	0.00	180.0	1
MB	OM	MD	OG	0.00	0.0	1	0.00	0.0	1	0.00	0.0	1
MB	OM	MD	OH	0.00	180.0	1	0.00	180.0	1	0.00	180.0	1

Table S7: Continuation #2 of Table S5.

Atom 1	Atom 2	Atom 3	Atom 4	— [Mn^{IV}_4] — V_i	γ_i	n_i	— [$Mn^{III}Mn^{IV}_3$] — V_i	γ_i	n_i	— [$Mn^{III}_2Mn^{IV}_2$] — V_i	γ_i	n_i
MC	OJ	MA	o	0.00	0.0	1	0.00	0.0	1	0.00	0.0	1
MC	OJ	MA	OE	0.00	180.0	1	0.00	180.0	1	0.00	180.0	1
MC	OJ	MA	OF	0.00	180.0	1	0.00	180.0	1	0.00	180.0	1
MC	OJ	MD	o	0.00	0.0	1	0.00	0.0	1	0.00	0.0	1
MC	OJ	MD	OG	0.00	180.0	1	0.00	180.0	1	0.00	180.0	1
MC	OJ	MD	OH	0.00	180.0	1	0.00	180.0	1	0.00	180.0	1
MC	OL	MA	o	0.00	180.0	1	0.00	180.0	1	0.00	180.0	1
MC	OL	MA	OE	0.00	180.0	1	0.00	180.0	1	0.00	180.0	1
MC	OL	MA	OF	0.00	0.0	1	0.00	0.0	1	0.00	0.0	1
MC	OL	MB	ON	0.00	0.0	1	0.00	0.0	1	0.00	0.0	1
MC	OL	MB	OO	0.00	180.0	1	0.00	180.0	1	0.00	180.0	1
MC	OL	MB	OP	0.00	180.0	1	0.00	180.0	1	0.00	180.0	1
MC	OM	MB	ON	0.00	180.0	1	0.00	180.0	1	0.00	180.0	1
MC	OM	MB	OO	0.00	180.0	1	0.00	180.0	1	0.00	180.0	1
MC	OM	MB	OP	0.00	0.0	1	0.00	0.0	1	0.00	0.0	1
MC	OM	MD	o	0.00	180.0	1	0.00	180.0	1	0.00	180.0	1
MC	OM	MD	OG	0.00	0.0	1	0.00	0.0	1	0.00	0.0	1
MC	OM	MD	OH	0.00	180.0	1	0.00	180.0	1	0.00	180.0	1
MD	OJ	MA	o	0.00	0.0	1	0.00	0.0	1	0.00	0.0	1
MD	OJ	MA	OE	0.00	180.0	1	0.00	180.0	1	0.00	180.0	1
MD	OJ	MA	OF	0.00	180.0	1	0.00	180.0	1	0.00	180.0	1
MD	OJ	MC	o	0.00	0.0	1	0.00	0.0	1	0.00	0.0	1
MD	OJ	MC	OD	0.00	180.0	1	0.00	180.0	1	0.00	180.0	1
MD	OJ	MC	OI	0.00	180.0	1	0.00	180.0	1	0.00	180.0	1
MD	OK	MA	o	0.00	180.0	1	0.00	180.0	1	0.00	180.0	1
MD	OK	MA	OE	0.00	0.0	1	0.00	0.0	1	0.00	0.0	1
MD	OK	MA	OF	0.00	180.0	1	0.00	180.0	1	0.00	180.0	1
MD	OK	MB	ON	0.00	180.0	1	0.00	180.0	1	0.00	180.0	1
MD	OK	MB	OO	0.00	0.0	1	0.00	0.0	1	0.00	0.0	1
MD	OK	MB	OP	0.00	180.0	1	0.00	180.0	1	0.00	180.0	1
MD	OM	MB	ON	0.00	180.0	1	0.00	180.0	1	0.00	180.0	1
MD	OM	MB	OO	0.00	180.0	1	0.00	180.0	1	0.00	180.0	1
MD	OM	MB	OP	0.00	0.0	1	0.00	0.0	1	0.00	0.0	1
MD	OM	MC	o	0.00	180.0	1	0.00	180.0	1	0.00	180.0	1
MD	OM	MC	OD	0.00	0.0	1	0.00	0.0	1	0.00	0.0	1
MD	OM	MC	OI	0.00	180.0	1	0.00	180.0	1	0.00	180.0	1
MA	OE	VB	OB	0.10	45.0	1	0.10	45.0	1	0.10	45.0	1
MA	OE	VB	OC	0.10	45.0	1	0.10	45.0	1	0.10	45.0	1
MA	OE	VB	OD	0.10	45.0	1	0.10	45.0	1	0.10	45.0	1
MA	OF	VC	OG	0.10	-45.0	1	0.10	-45.0	1	0.10	-45.0	1
MA	OF	VC	OQ	0.10	-45.0	1	0.10	-45.0	1	0.10	-45.0	1
MA	OF	VC	OR	0.10	-45.0	1	0.10	-45.0	1	0.10	-45.0	1
MC	OD	VB	OB	0.10	-45.0	1	0.10	-45.0	1	0.10	-45.0	1
MC	OD	VB	OC	0.10	-45.0	1	0.10	-45.0	1	0.10	-45.0	1
MC	OD	VB	OE	0.10	-45.0	1	0.10	-45.0	1	0.10	-45.0	1
MC	OI	VB	OB	0.10	45.0	1	0.10	45.0	1	0.10	45.0	1
MC	OI	VB	OC	0.10	45.0	1	0.10	45.0	1	0.10	45.0	1
MC	OI	VB	OH	0.10	45.0	1	0.10	45.0	1	0.10	45.0	1
MD	OG	VC	OF	0.10	45.0	1	0.10	45.0	1	0.10	45.0	1
MD	OG	VC	OQ	0.10	45.0	1	0.10	45.0	1	0.10	45.0	1
MD	OG	VC	OR	0.10	45.0	1	0.10	45.0	1	0.10	45.0	1
MD	OH	VB	OB	0.10	-45.0	1	0.10	-45.0	1	0.10	-45.0	1
MD	OH	VB	OC	0.10	-45.0	1	0.10	-45.0	1	0.10	-45.0	1
MD	OH	VB	OI	0.10	-45.0	1	0.10	-45.0	1	0.10	-45.0	1

Table S8: Continuation #3 of Table S5.

Atom 1	Atom 2	Atom 3	Atom 4	— $[Mn^{IV}_4]$ — V_i	γ_i	n_i	— $[Mn^{III}Mn^{IV}_3]$ — V_i	γ_i	n_i	— $[Mn^{III}_2Mn^{IV}_2]$ — V_i	γ_i	n_i
VB	OE	MA	o	0.20	-90.0	1	0.20	-90.0	1	0.20	-90.0	1
VB	OE	MA	OF	0.20	-90.0	1	0.20	-90.0	1	0.20	-90.0	1
VB	OE	MA	OJ	0.20	-90.0	1	0.20	-90.0	1	0.20	-90.0	1
VB	OE	MA	OK	0.00	0.0	1	0.00	0.0	1	0.00	0.0	1
VB	OE	MA	OL	0.20	-90.0	1	0.20	-90.0	1	0.20	-90.0	1
VC	OF	MA	o	0.20	-90.0	1	0.20	-90.0	1	0.20	-90.0	1
VC	OF	MA	OE	0.20	90.0	1	0.20	90.0	1	0.20	90.0	1
VC	OF	MA	OJ	0.20	90.0	1	0.20	90.0	1	0.20	90.0	1
VC	OF	MA	OK	0.20	90.0	1	0.20	90.0	1	0.20	90.0	1
VC	OF	MA	OL	0.00	0.0	1	0.00	0.0	1	0.00	0.0	1
VB	OD	MC	o	0.20	90.0	1	0.20	90.0	1	0.20	90.0	1
VB	OD	MC	OI	0.20	90.0	1	0.20	90.0	1	0.20	90.0	1
VB	OD	MC	OJ	0.20	90.0	1	0.20	90.0	1	0.20	90.0	1
VB	OD	MC	OL	0.20	90.0	1	0.20	90.0	1	0.20	90.0	1
VB	OD	MC	OM	0.00	0.0	1	0.00	0.0	1	0.00	0.0	1
VB	OI	MC	o	0.20	90.0	1	0.20	90.0	1	0.20	90.0	1
VB	OI	MC	OD	0.20	90.0	1	0.20	90.0	1	0.20	90.0	1
VB	OI	MC	OJ	0.20	-90.0	1	0.20	-90.0	1	0.20	-90.0	1
VB	OI	MC	OL	0.00	0.0	1	0.00	0.0	1	0.00	0.0	1
VB	OI	MC	OM	0.20	-90.0	1	0.20	-90.0	1	0.20	-90.0	1
VB	OH	MD	o	0.20	90.0	1	0.20	90.0	1	0.20	90.0	1
VB	OH	MD	OG	0.20	90.0	1	0.20	90.0	1	0.20	90.0	1
VB	OH	MD	OJ	0.20	90.0	1	0.20	90.0	1	0.20	90.0	1
VB	OH	MD	OK	0.00	0.0	1	0.00	0.0	1	0.00	0.0	1
VB	OH	MD	OM	0.20	90.0	1	0.20	90.0	1	0.20	90.0	1
VC	OG	MD	o	0.20	-90.0	1	0.20	-90.0	1	0.20	-90.0	1
VC	OG	MD	OH	0.20	90.0	1	0.20	90.0	1	0.20	90.0	1
VC	OG	MD	OJ	0.20	-90.0	1	0.20	-90.0	1	0.20	-90.0	1
VC	OG	MD	OK	0.20	-90.0	1	0.20	-90.0	1	0.20	-90.0	1
VC	OG	MD	OM	0.00	0.0	1	0.00	0.0	1	0.00	0.0	1
VA	OB	VB	OC	0.80	0.0	1	0.80	0.0	1	0.80	0.0	1
VA	OB	VB	OD	0.80	0.0	1	0.80	0.0	1	0.80	0.0	1
VA	OB	VB	OE	0.80	0.0	1	0.80	0.0	1	0.80	0.0	1
VA	OB	VB	OH	0.80	0.0	1	0.80	0.0	1	0.80	0.0	1
VA	OB	VB	OI	0.80	0.0	1	0.80	0.0	1	0.80	0.0	1
VA	OR	VC	OF	0.80	0.0	1	0.80	0.0	1	0.80	0.0	1
VA	OR	VC	OG	0.80	0.0	1	0.80	0.0	1	0.80	0.0	1
VA	OR	VC	OQ	0.80	0.0	1	0.80	0.0	1	0.80	0.0	1
VB	OB	VA	OA	0.80	0.0	1	0.80	0.0	1	0.80	0.0	1
VB	OB	VA	OB	0.80	0.0	1	0.80	0.0	1	0.80	0.0	1
VB	OB	VA	OR	0.80	0.0	1	0.80	0.0	1	0.80	0.0	1
VC	OR	VA	OA	0.80	0.0	1	0.80	0.0	1	0.80	0.0	1
VC	OR	VA	OB	0.80	0.0	1	0.80	0.0	1	0.80	0.0	1

Table S9: Continuation #4 of Table S5.

Atom 1	Atom 2	Atom 3	Atom 4	— $[Mn^{IV}_4]$ — V_i	γ_i	n_i	— $[Mn^{III}Mn^{IV}_3]$ — V_i	γ_i	n_i	— $[Mn^{III}_2Mn^{IV}_2]$ — V_i	γ_i	n_i
c	o	MA	OE	0.00	10.95	1	3.00	8.03	1	3.00	8.03	1
c	o	MA	OF	0.00	40.90	1	3.00	8.03	1	3.00	8.03	1
c	o	MA	OJ	0.00	10.95	1	1.50	10.95	1	1.50	10.95	1
c	o	MA	OK	0.00	40.90	1	1.50	40.90	1	1.50	40.90	1
c	o	MA	OL	0.00	40.90	1	3.00	8.03	1	3.00	8.03	1
c	o	MC	OD	0.00	131.90	1	1.50	131.90	1	1.50	131.90	1
c	o	MC	OI	0.00	131.90	1	1.50	131.90	1	1.50	131.90	1
c	o	MC	OJ	0.00	10.95	1	1.50	10.95	1	1.50	10.95	1
c	o	MC	OL	0.00	40.90	1	3.00	8.03	1	3.00	8.03	1
c	o	MC	OM	0.00	40.90	1	3.00	8.03	1	3.00	8.03	1
c	o	MD	OG	0.00	131.90	1	3.00	8.03	1	3.00	8.03	1
c	o	MD	OH	0.00	131.90	1	3.00	8.03	1	3.00	8.03	1
c	o	MD	OJ	0.00	10.95	1	1.50	10.95	1	1.50	10.95	1
c	o	MD	OK	0.00	40.90	1	1.50	40.90	1	1.50	40.90	1
c	o	MD	OM	0.00	40.90	1	3.00	8.03	1	3.00	8.03	1
c	ON	MB	OK	0.00	37.15	1	1.50	37.15	1	1.50	37.15	1
c	ON	MB	OL	0.00	37.15	1	3.00	8.03	1	3.00	8.03	1
c	ON	MB	OM	0.00	37.15	1	3.00	8.03	1	3.00	8.03	1
c	ON	MB	OO	0.00	131.47	1	1.50	131.76	1	1.50	131.76	1
c	ON	MB	OP	0.00	131.47	1	1.50	131.76	1	1.50	131.76	1
c	OO	MB	OK	0.00	37.15	1	1.50	37.15	1	1.50	37.15	1
c	OO	MB	OL	0.00	37.15	1	3.00	8.03	1	3.00	8.03	1
c	OO	MB	OM	0.00	37.15	1	3.00	8.03	1	3.00	8.03	1
c	OO	MB	ON	0.00	131.47	1	1.50	131.76	1	1.50	131.76	1
c	OO	MB	OP	0.00	131.47	1	1.50	131.76	1	1.50	131.76	1
c	OP	MB	OK	0.00	37.15	1	1.50	37.15	1	1.50	37.15	1
c	OP	MB	OL	0.00	37.15	1	3.00	8.03	1	3.00	8.03	1
c	OP	MB	OM	0.00	37.15	1	3.00	8.03	1	3.00	8.03	1
c	OP	MB	ON	0.00	131.47	1	1.50	131.76	1	1.50	131.76	1
c	OP	MB	OO	0.00	131.47	1	1.50	131.76	1	1.50	131.76	1
MA	o	c	c3	0.00	176.11	2	2.80	176.11	2	2.80	176.11	2
MB	ON	c	c3	0.00	175.11	2	2.80	175.11	2	2.80	175.11	2
MB	OO	c	c3	0.00	175.11	2	2.80	175.11	2	2.80	175.11	2
MB	OP	c	c3	0.00	175.11	2	2.80	175.11	2	2.80	175.11	2
MC	o	c	c3	0.00	176.11	2	2.80	176.11	2	2.80	176.11	2
MD	o	c	c3	0.00	176.11	2	2.80	176.11	2	2.80	176.11	2
MB	ON	c	o	0.00	4.89	2	2.80	4.89	2	2.80	4.89	2
MB	OO	c	o	0.00	4.89	2	2.80	4.89	2	2.80	4.89	2
MB	OP	c	o	0.00	4.89	2	2.80	4.89	2	2.80	4.89	2
MD	o	c	ON	0.00	4.09	2	2.80	4.09	2	2.80	4.09	2
MC	o	c	OO	0.00	4.09	2	2.80	4.09	2	2.80	4.09	2
MA	o	c	OP	0.00	4.09	2	2.80	4.09	2	2.80	4.09	2
hc	c3	c	o	0.83	0.00	1	0.83	0.00	1	0.83	0.00	1
hc	c3	c	ON	0.83	0.00	1	0.83	0.00	1	0.83	0.00	1
hc	c3	c	OO	0.83	0.00	1	0.83	0.00	1	0.83	0.00	1
hc	c3	c	OP	0.83	0.00	1	0.83	0.00	1	0.83	0.00	1

Table S10: List of all obtained parameters for the **Lennard-Jones interaction**. Parameters ε_i are in kcal mol^{-1} and $r_{i,\text{min}}$ in Å.

	— $[Mn^{IV}_4]$ —		— $[Mn^{III}Mn^{IV}_3]$ —		— $[Mn^{III}_2Mn^{IV}_2]$ —	
Atom 1	$r_{i,\text{min}}$	ε_i	$r_{i,\text{min}}$	ε_i	$r_{i,\text{min}}$	ε_i
MA	1.4670	0.0296	1.4670	0.0296	1.4670	0.0296
MB	1.4670	0.0296	1.4670	0.0296	1.4670	0.0296
MC	1.4670	0.0296	1.4670	0.0296	1.4670	0.0296
MD	1.4670	0.0296	1.4670	0.0296	1.4670	0.0296
OA	1.6562	0.1700	1.6562	0.1700	1.6562	0.1700
OB	1.6562	0.1700	1.6562	0.1700	1.6562	0.1700
OC	1.6562	0.1700	1.6562	0.1700	1.6562	0.1700
OD	1.6562	0.1700	1.6562	0.1700	1.6562	0.1700
OE	1.6562	0.1700	1.6562	0.1700	1.6562	0.1700
OF	1.6562	0.1700	1.6562	0.1700	1.6562	0.1700
OG	1.6562	0.1700	1.6562	0.1700	1.6562	0.1700
OI	1.6562	0.1700	1.6562	0.1700	1.6562	0.1700
OJ	1.6562	0.1700	1.6562	0.1700	1.6562	0.1700
OK	1.6562	0.1700	1.6562	0.1700	1.6562	0.1700
OL	1.6562	0.1700	1.6562	0.1700	1.6562	0.1700
OM	1.6562	0.1700	1.6562	0.1700	1.6562	0.1700
ON	1.6562	0.1700	1.6562	0.1700	1.6562	0.1700
OO	1.6562	0.1700	1.6562	0.1700	1.6562	0.1700
OP	1.6562	0.1700	1.6562	0.1700	1.6562	0.1700
OQ	1.6562	0.1700	1.6562	0.1700	1.6562	0.1700
OR	1.6562	0.1700	1.6562	0.1700	1.6562	0.1700
VA	1.5720	0.0160	1.5720	0.0160	1.5720	0.0160
VB	1.5720	0.0160	1.5720	0.0160	1.5720	0.0160
VC	1.5720	0.0160	1.5720	0.0160	1.5720	0.0160
Na+	1.3690	0.0874	1.3690	0.0874	1.3690	0.0874

Table S11: List of all obtained parameters for the **Coulomb interaction**. Parameters q_i are in multiples of the elementary charge.

		— [Mn_4^{IV}] —	— [$Mn^{III}Mn_3^{IV}$] —	— [$Mn_2^{III}Mn_2^{IV}$] —
Atom	Atom type	q_i	q_i	q_i
Mn1	MA	+1.4898	+1.5854	+1.5188
O1	OK	-0.8018	-0.8319	-0.8742
Mn2	MB	+1.6073	+1.5404	+1.4504
O2	OL	-0.8034	-0.8463	-0.8570
Mn3	MC	+1.5017	+1.4749	+1.6400
O4	OM	-0.8034	-0.8463	-0.8570
Mn4	MD	+1.4898	+1.5854	+1.5188
O3	OJ	-1.0519	-1.0611	-1.0900
O20	OQ	-0.4930	-0.5640	-0.6708
V4	VC	+1.2944	+1.3610	+1.4209
O21	OF	-0.6254	-0.6831	-0.7685
O22	OG	-0.6254	-0.6831	-0.7685
O23	OR	-0.6408	-0.6907	-0.7324
V3	VA	+1.3358	+1.3972	+1.4505
O17	OA	-0.4869	-0.5625	-0.6322
O18	OB	-0.6435	-0.6912	-0.7288
V2	VB	+1.3030	+1.3645	+1.4205
O14	OH	-0.6274	-0.6848	-0.7621
O15	OI	-0.6294	-0.7198	-0.7321
O16	OC	-0.4944	-0.5802	-0.6493
O12	OC	-0.4944	-0.5802	-0.6493
V1	VB	+1.3030	+1.3645	+1.4205
O11	OD	-0.6294	-0.7198	-0.7321
O13	OE	-0.6274	-0.6848	-0.7621
O19	OB	-0.6435	-0.6912	-0.7288

		— [Mn_4^{IV}] —	— [$Mn^{III}Mn_3^{IV}$] —	
Atom	Atom type	q_i	q_i	q_i
C2	c3	-0.5936	-0.5939	-0.6022
H1	hc	+0.1714	+0.1489	+0.1284
H2	hc	+0.1714	+0.1489	+0.1284
H3	hc	+0.1714	+0.1489	+0.1284
C1	c	+0.8877	+0.9074	+0.9573
O6	ON	-0.6348	-0.6390	-0.6668
O5	o	-0.5746	-0.6180	-0.7049
C3	c3	-0.5936	-0.5939	-0.6022
H4	hc	+0.1714	+0.1489	+0.1284
H5	hc	+0.1714	+0.1489	+0.1284
H6	hc	+0.1714	+0.1489	+0.1284
C4	c	+0.8963	+0.9335	+0.9320
O8	OO	-0.6392	-0.6730	-0.6484
O7	o	-0.5787	-0.6724	-0.6493
C5	c3	-0.5936	-0.5939	-0.6022
H7	hc	+0.1714	+0.1489	+0.1284
H8	hc	+0.1714	+0.1489	+0.1284
H9	hc	+0.1714	+0.1489	+0.1284
C6	c	+0.8877	+0.9074	+0.9573
O9	o	-0.5746	-0.6180	-0.7049
O10	OP	-0.6348	-0.6390	-0.6668

S2 Densities of Acetonitrile/Water Mixtures

To select an acetonitrile/water force field combination, we compared simulated average densities versus experiment[22,23] across varying molar fractions of acetonitrile $x(CH_3CN)$ and water models. Table S12 shows the densities ρ obtained from MD simulations applying the respective water models and molar fraction of acetronitrile x (CH_3CN).

Table S12: Densities ρ of the respective water model and molar fraction x (CH_3CN).

		ρ (g/cm^3)		
water model	x (CH_3CN)	def2-svp, b3lyp	HF, def2-svp	six-site[12]
TIP3P	0.0	0.98	0.98	0.98
	0.25	0.85	0.87	0.88
	0.5	0.79	0.81	0.83
	0.75	0.75	0.77	0.79
	1.0	0.73	0.75	0.77
SPC/Fw	0.0	1.01	1.01	1.01
	0.25	0.87	0.89	0.91
	0.5	0.80	0.82	0.84
	0.75	0.76	0.78	0.80
	1.0	0.73	0.75	0.77
q-SPC/Fw	0.0	1.03	1.03	1.03
	0.25	0.88	0.91	0.93
	0.5	0.81	0.83	0.85
	0.75	0.76	0.78	0.81
	1.0	0.73	0.75	0.77

Figure S1 displays the densities, averaged over the course of the MD simulation, as a function of x(CH_3CN) for different force field parameters.

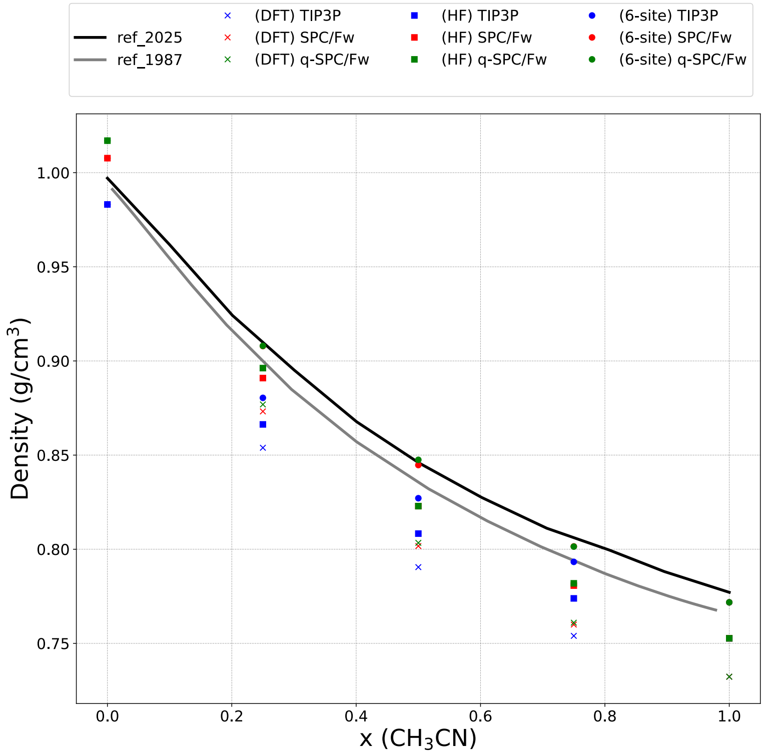

Figure S2: Density obtained from MD simulations plotted against the molar fraction x (CH_3CN). Experimental references densities are represented in solid lines.[22,23]

For pure water (x(CH_3CN)=0.0), both the flexible SPC/Fw water model and TIP3P exhibit similarly good agreement with the experimental data. The SPC/Fw model deviates by approximately +0.02 g/cm^3, while TIP3P deviates by around -0.02 g/cm^3 from the experimental reference. For pure acetonitrile (x(CH_3CN)=1.0), the best agreement with experimental data is observed for the six-site acetonitrile model, followed by the HF- and DFT-parametrized force fields. In the case of mixtures, the six-site model combined with the SPC/Fw and q-SPC/Fw water model consistently demonstrate excellent agreement with the experimental values. Based on this, we used the six-site CH_3CN with SPC/Fw for all subsequent POM simulations.

S3 Radial distribution functions between solvents and individual atoms in {MnV}$^{n-}$ in pure solvents

In this work, we adopt the same atom notation as used in our previous work.[24] Figure S3 shows the three-dimensional structure of $(nBu_4N)_3[Mn^{III}{}_2Mn^{IV}{}_2V_4O_{17}(OAc)_3]$, including the atom labels and the indicated Jahn–Teller axes (in green).

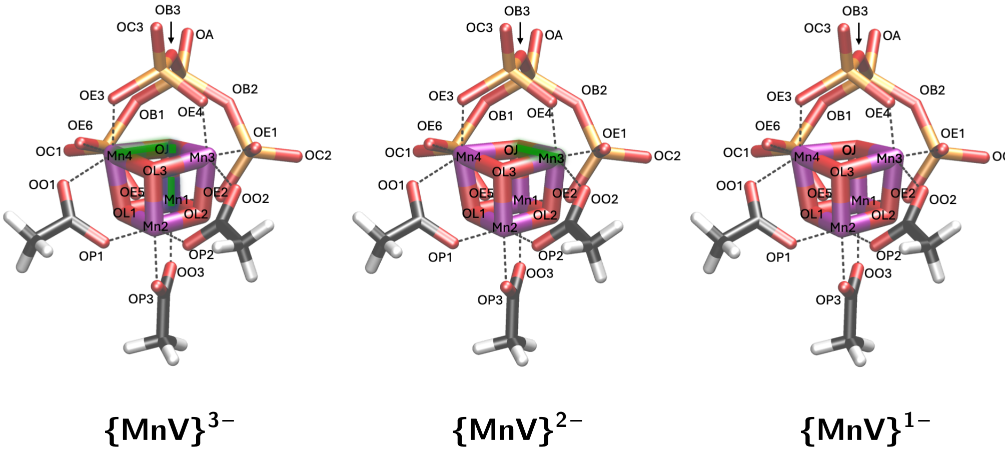

Figure S3: Three-dimensional structure of $[(Mn_4O_4)(V_4O_{13})(OAc)_3]^{n-}$. OA and OC denote terminal oxygen atoms; OB and OE are bridging oxygens; OL and OJ are cubane-site oxygens; OO and OP correspond to acetate oxygens. Jahn-Teller axes are highlighted in green.

Figure S4 and S5 show site-specific RDFs, calculated between the water hydrogen atoms (Figure S4) and hydrogen acetonitrile atoms (Figure S5) and all 23 oxygen atoms and 4 manganese atoms in {MnV}$^{\mathbf{n-}}$.

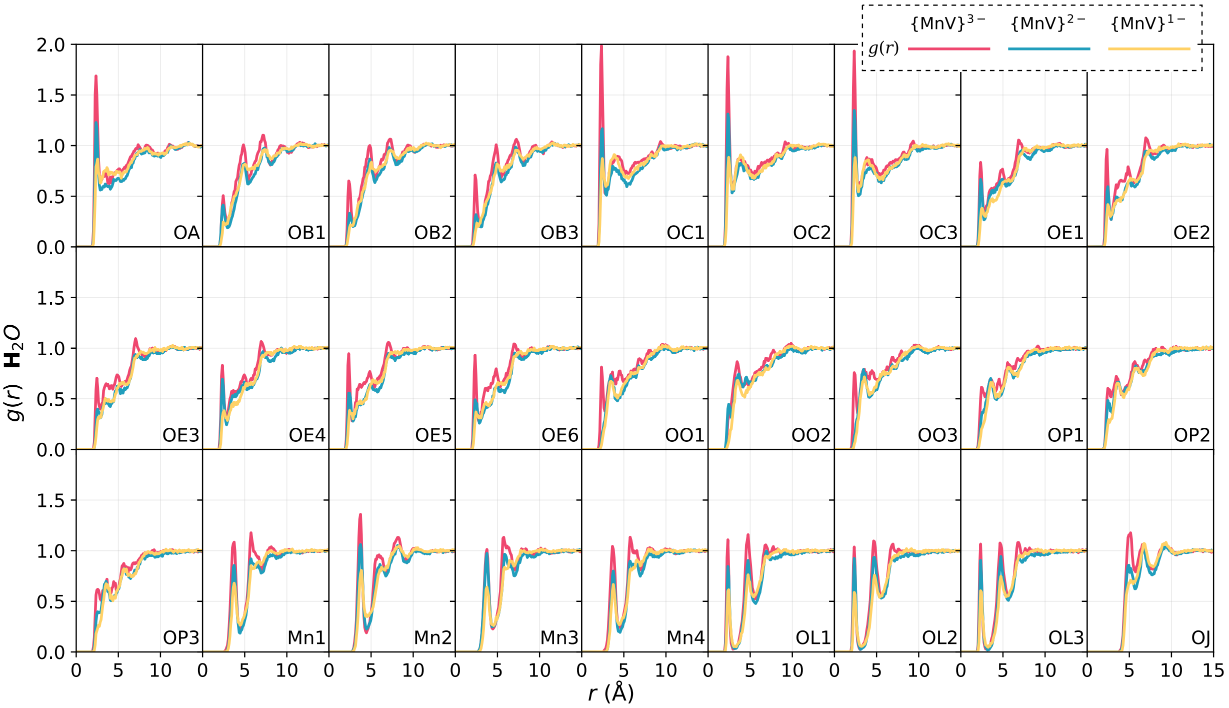

Figure S4: Radial distribution functions (RDFs) between the center of mass (CoM) of {MnV}$^{\mathbf{n-}}$ and the hydrogen atoms of water in pure water.

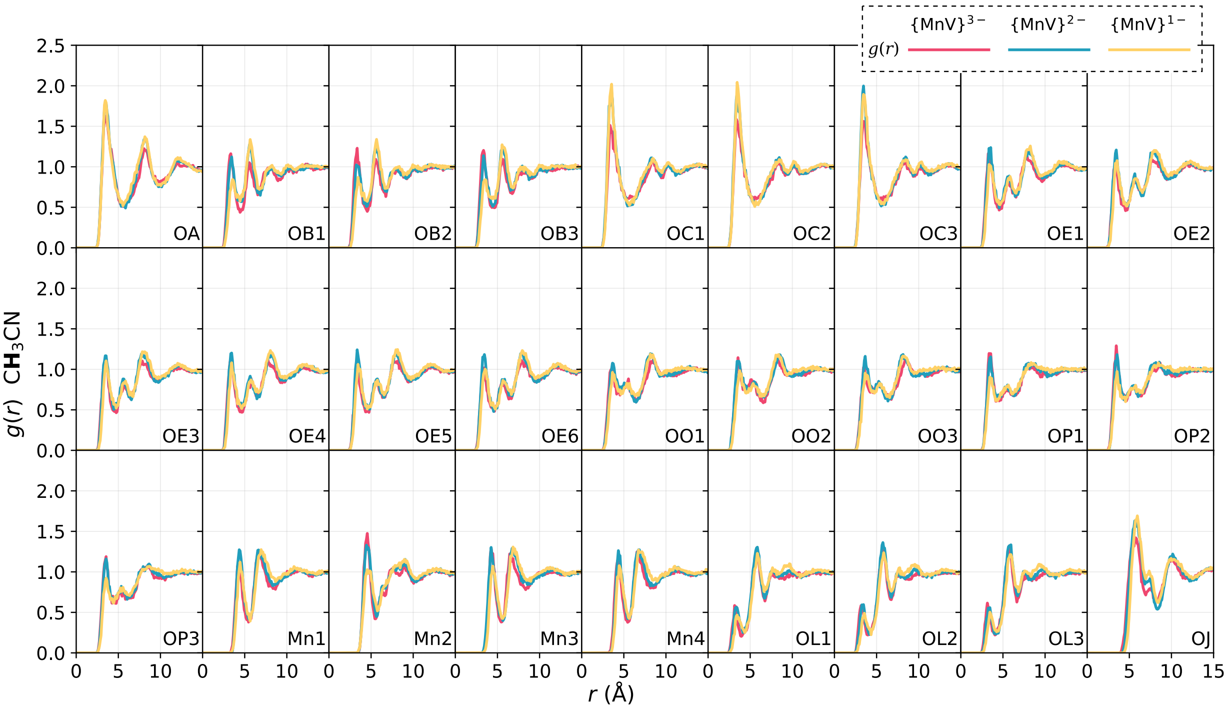

Figure S5: RDFs between the center of mass (CoM) of $\{MnV\}^{n-}$ and the hydrogen atoms of acetonitrile in pure acetonitrile.

Reduction of MnV^{n-} induces Jahn–Teller distortion in the cubane core, as discussed elsewhere.[24] This geometric asymmetry is also reflected in the corresponding RDFs. For instance, small but noticeable variations in the $g(r)$ values of $\{MnV\}^{3-}$ for the bridging OB oxygen atoms (Figure S4) suggest that certain sites are slightly displaced outward, making them more accessible to the surrounding solvent.

S4 Radial distribution functions between center of mass of {MnV}n- and solvent molecules (water and acetonitrile)

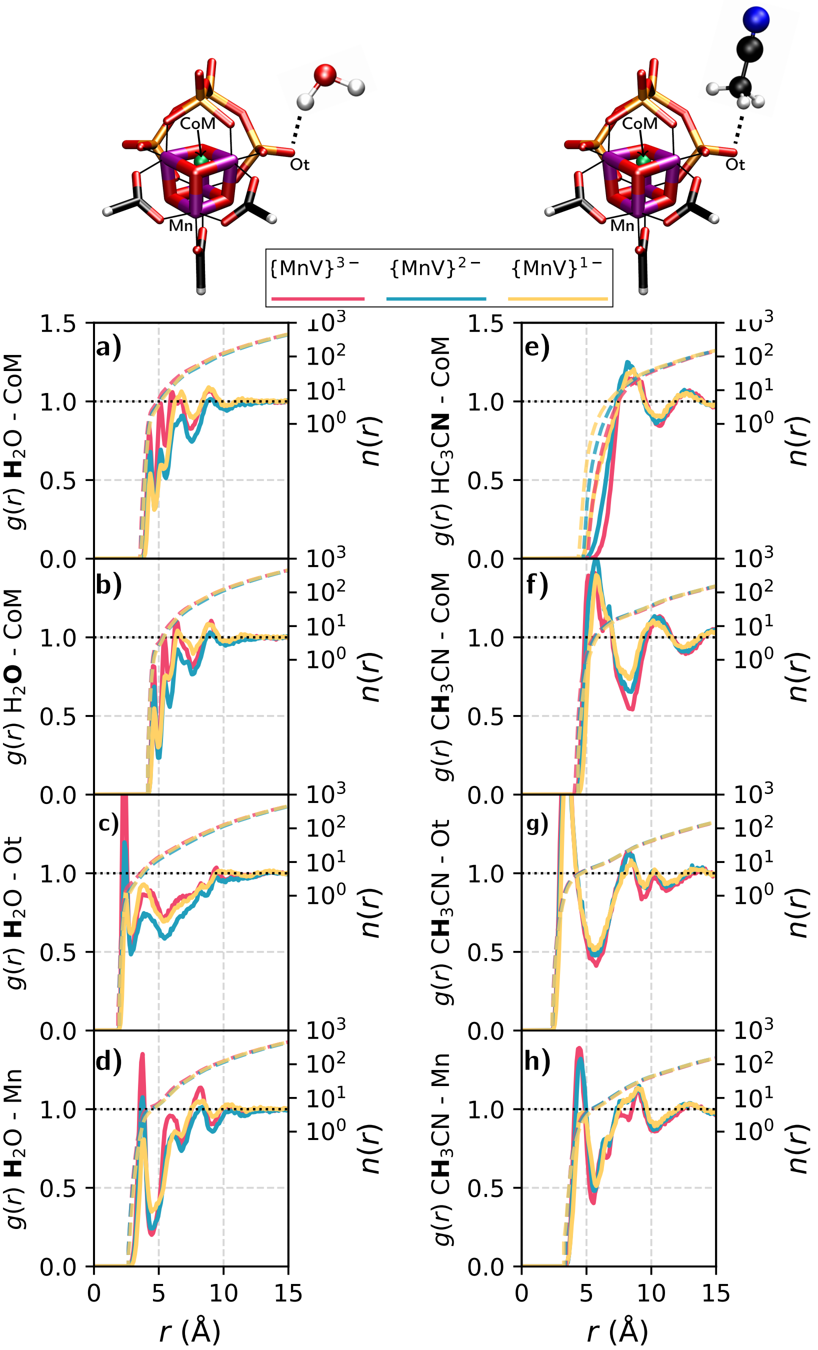

Figure S6: Radial distribution functions, $g(r)$, of the $\{MnV\}^{n-}$ in pure water (left) and pure acetonitrile (right). Functions are plotted between specific atoms of the solvent and either (panels a,b,e,f) the center of mass (CoM) of the cluster, or (c,g) the terminal oxygen of the cluster, denoted Ot (OC1 in Figure S3) or (d,h) the apical Mn atom of the cubane (Mn2 in Figure S3). For illustration, a green dummy atom is placed at the CoM of the $\{MnV\}^{n-}$ cluster. Three-dimensional renderings of $\{MnV\}^{n-}$ including an example of the most frequent solvent molecule were generated with VMD.[25] Dashed lines indicate the integrated solvation numbers $n(r)$.

S5 Radial distribution functions between solvents and individual atoms in {MnV}$^{n-}$ in acetonitrile/water mixtures (5 vol% water)

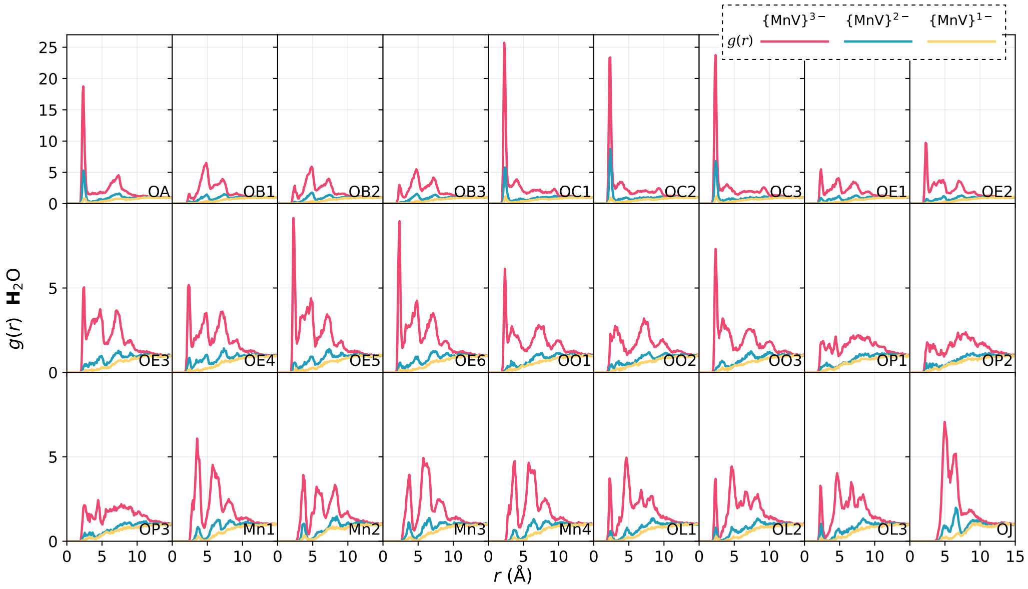

Figure S7: RDFs between the center of mass (CoM) of {MnV}$^{n-}$ and the hydrogen atoms of water in a acetonitrile/water mixture (5 vol% water).

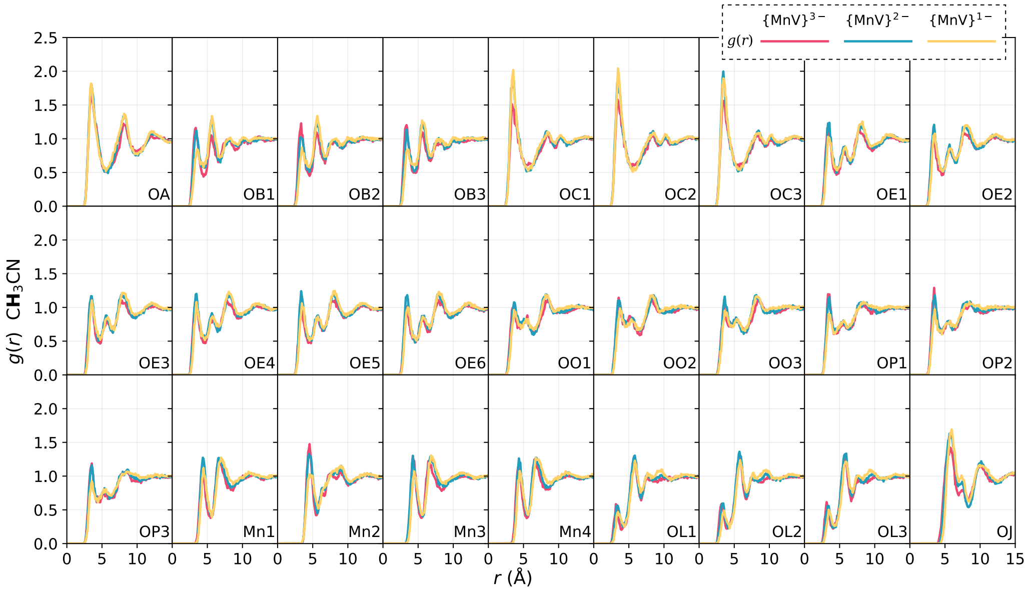

Figure S8: RDFs between the center of mass (CoM) of {MnV}$^{n-}$ and the hydrogen atoms of acetonitrile in a acetonitrile/water mixture (5 vol% water).

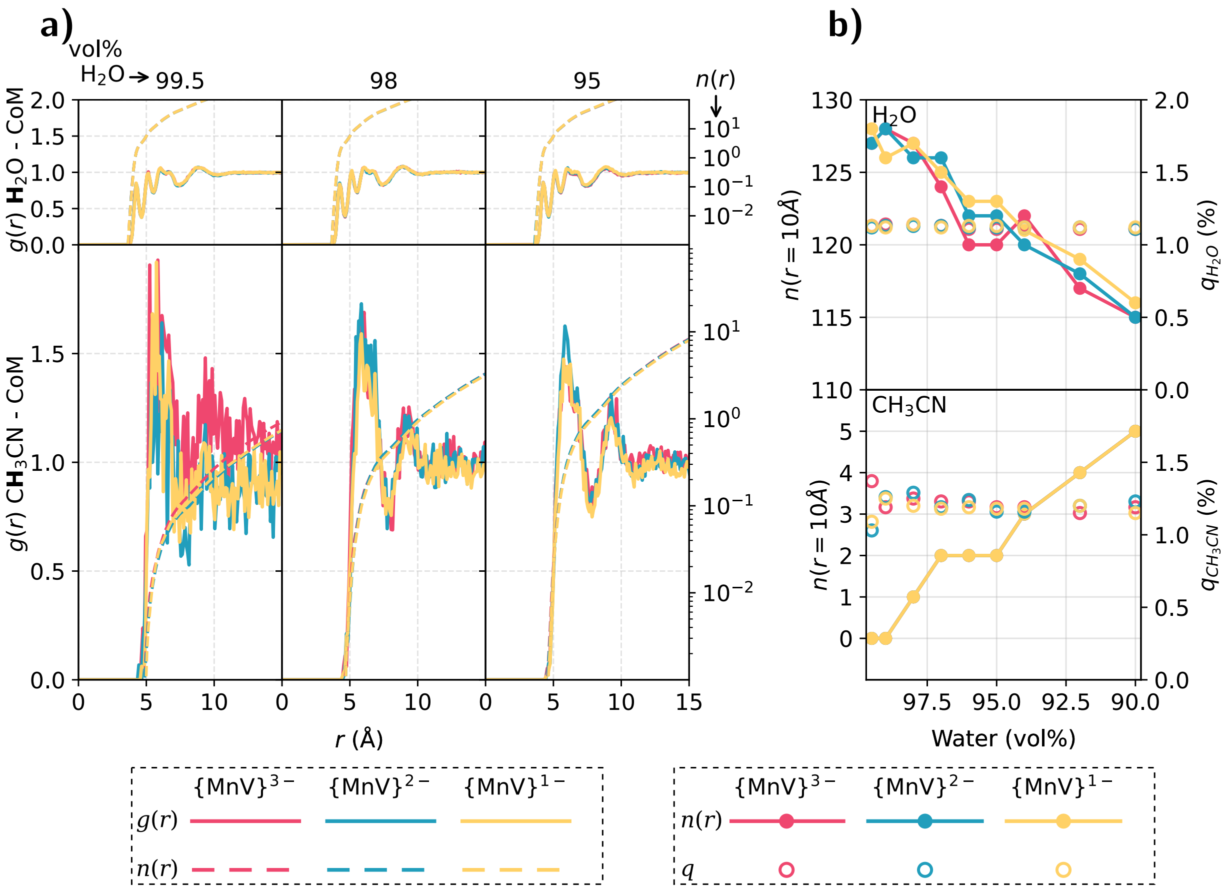

Figure S9: a) RDFs, $g(r)$, between the center of mass (CoM) of and the hydrogen atoms of water and acetonitrile. Dashed lines indicate the corresponding integrated solvation numbers $n(r)$. b) Integrated solvation numbers $n(r)$ at $r = 10$ Å, are shown as solid lines with filled markers at the data points, while the solvent fraction q (fraction of solvent molecules residing within 10 Å of the catalyst) is shown as hollow circular markers. Both quantities are plotted as a function of water content.

For water, the radial distribution functions $g(r)$ show no significant differences across the varying water contents or oxidation states. In contrast, the integrated solvation numbers $N(r)$ reveal pronounced variations in microsolvation upon decreasing the water content. In particular, a steep decrease in $N(r)$ is observed for MnV^{n-}, highlighting a strong dependence on the oxidation state. While systems containing 0.5, 2, and 5 vol% water exhibit a difference in $N(r)$ across the oxidation states, this effect is substantially reduced at higher water contents. These results indicate that systems with low water concentrations are considerably more sensitive to the catalyst charge than nearly fully aqueous solutions.

In contrast to water, acetonitrile exhibits no significant changes in $g(r)$ with either oxidation state or water content. Although a gradual increase in $N(r)$ is observed, this trend can be attributed to the increasing number of acetonitrile molecules in the system rather than to preferential interactions with the catalyst. This interpretation is supported by the nearly constant values of q_{CH_3CN} across all water contents, underscoring the weak sensitivity of acetonitrile solvation to both the oxidation state of the catalyst and the solvent composition when compared to water.

S6 Hydrogen bonds in acetonitrile/water mixtures (5 vol% water)

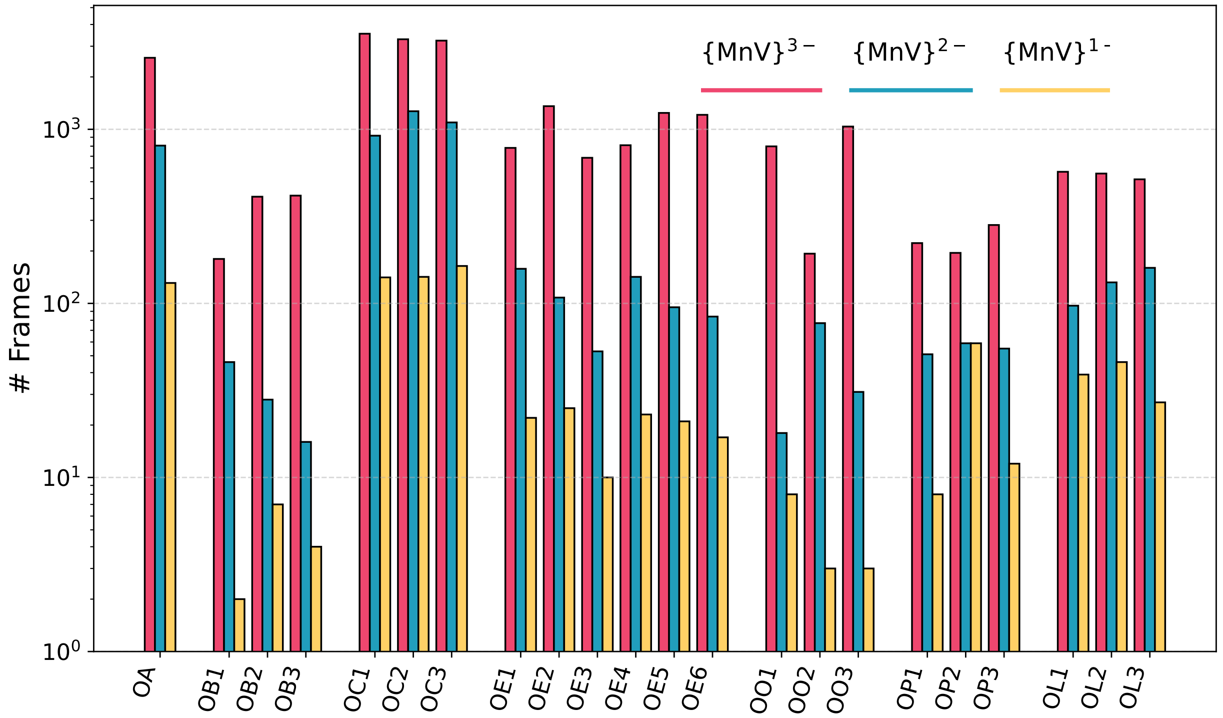

Figure S10: Computed number of frames in which a hydrogen bond is formed between a solvent hydrogen atom and any of the 23 oxygen sites of {MnV}$^{\mathbf{n-}}$. The analysis was performed using CPPTRAJ.[19] All {MnV}$^{\mathbf{n-}}$ species were solvated in an acetonitrile/water mixture containing 5 vol% water.

To gain deeper insight into the interactions between the catalyst and water, we computed the number of hydrogen bonds formed at each oxygen site of the catalyst (Section S7). Regardless the charge, the majority of hydrogen bonds are located at the terminal oxygen sites, as expected, since these sites are most frequently surrounded by solvent water molecules. The number of hydrogen bonds formed does not differ significantly across the individual terminal oxygen sites of the cluster.

For {MnV}$^{\mathbf{1-}}$, the cubane core participates in more hydrogen bonds compared to the acetate ligands or bridging oxygen sites. For the more negative charge states, Jahn-Teller distortion of the catalyst leads to deformation of the cubane core. This also introduces an asymmetry in the tendency of hydrogen bonds to form. By opening the cubane core, terminal oxygen sites become more accessible to water. Additionally, the likelihood of the cubane core participating in hydrogen bonding appears to decrease upon reduction. This is likely due to the formation of a strong hydrogen-bonding network around the terminal oxygen sites, which hinders water molecules from interacting with the cubane core. This finding contrasts with our earlier work, where QM calculations indicated that the cubane core is the preferred site of protonation upon reduction.[24] However, those calculations did not account for explicit solvent interactions, thereby neglecting the possible formation of a solvation shell or steric effects that could hinder protonation of the cubane core.

S7 Instrumentation

Attenuated total reflectance-Fourier-transformed infrared spectroscopy (ATR-FT-IR) were performed using a Bruker Alpha II equipped with an ATR Platinum Diamond unit. The data were recorded with 24 scans at a resolution of 4 cm^{-1}. All spectra were background-corrected within the Bruker OPUS 8.1 program suite.

Liquid Fourier-transformed infrared spectroscopy (liquid FT-IR) measurements were performed using a Bruker Alpha II equipped with Alpha II-T sampling module. The data were recorded with 16 scans at a resolution of 2 cm^{-1} in an Omnis liquid cell with CaF_2 windows at a path length of 0.2 mm. All spectra were background-corrected within the Bruker OPUS 8.1 program suite using the solvent as reference.

High resolution electrospray ionization mass spectrometry (ESI MS) was carried out on an Agilent 6545 QTOF-HRAM-MS system in negative ion mode at a drying gas temperature of T = 180 °C.

Nuclear magnetic resonance (NMR) spectroscopy

Bruker Avance Neo NMR spectrometer (Bruker Biospin GmbH, Rheinstetten, Germany) equipped with a 5 mm BBF/F/H TBO iProbe head and Sample Case Plus autosampler was used to record all ^1H NMR spectra at ^1H frequency of 400.3 MHz. The ^1H spectra were recorded applying a 30° pulse averaging 16 scans with 2 s recycle delay. The ^1H T1 relaxation was measured using the inversion recovery pulse sequence averaging 16 scans with 5 s recycle delay and variable delays of 0.0000005 s, 0.000005 s, 0.00001 s, 0.0001 s, 0.0005 s, 0.001 s, 0.005 s, 0.01 s, 0.1 s, 0.25 s, 0.5 s and 1 s. The NMR spectra were analyzed using the program MestReNova (version 14.3.2-32681 / 23.03.2023, Mestrelab Re-search S. L.). The oxygen free samples were prepared by three freeze pump thaw cycles and sealed in the glovebox with Leica Cryptoseal (Leica Microsystems Vertrieb GmbH, Wetzlar, Germany). The ^1H NMR spectra were referenced using the solvent lock (2H) signal in accordance with the IUPAC recom-mended secondary referencing methods.[?] ^{51}V NMR spectra were referenced to external $VOCl_3$ + 5% C_6D_6 at 0 ppm.

UV/Vis/NIR spectroscopy was performed on a Cary 3500 UV/Vis/NIR spectrophotometer equipped with a Xenon flash lamp (250 Hz). Measurements were performed in quartz glass cuvettes (d = 10.0 mm).

Electrochemical Investigations

All experiments were carried out under ambient conditions in water free acetonitrile at room temperature, using 0.1 M nBu_4NPF_6 as supporting electrolyte. Cyclic voltammetry and square wave voltammetry were performed on a CH Instruments 760E potentiostat in three-electrode configuration: a glassy carbon with d = 3.0 mm was used as working electrode, a silver wire in acetonitrile containing 0.1 M nBu_4NPF_6 and 10 mM $AgNO_3$ was used as reference electrode and a platinum wire was used as counter electrode. All electrodes were cleaned with acetone and acetonitrile each time before use and the working electrode was additionally polished with 0.05 μm Al_2O_3 before each measurement. Chronoam-perometry was performed on a CH Instruments 760E potentiostat in three-electrode configuration: platinum wires were used as working and counter electrode, a silver wire in acetonitrile containing 0.1 M nBu_4NPF_6 and 10 mM $AgNO_3$ was used as a reference electrode. All electrodes were cleaned with acetone and acetonitrile before each experiment. During the experiment, the working electrode was kept at the defined potential while the solution was stirred vigorously. The electrolysis was stopped after 2000 s.

Chemicals

All chemical reagents were obtained commercially and used as received unless stated otherwise.

$(n\text{-}Bu_4N)_3[Mn_4V_4O_{17}(OAc)_3]$ *x* 3 H_2O (=$(n\text{-}Bu_4N)_3$MnV) was prepared according to the literature.[26]

The identity and purity of the brown cubic block shaped single crystals of $(n\text{-}Bu_4N)_3MnV$ was confirmed by FT-IR, ESI MS and NMR spectroscopy as well as cyclic voltammetry.

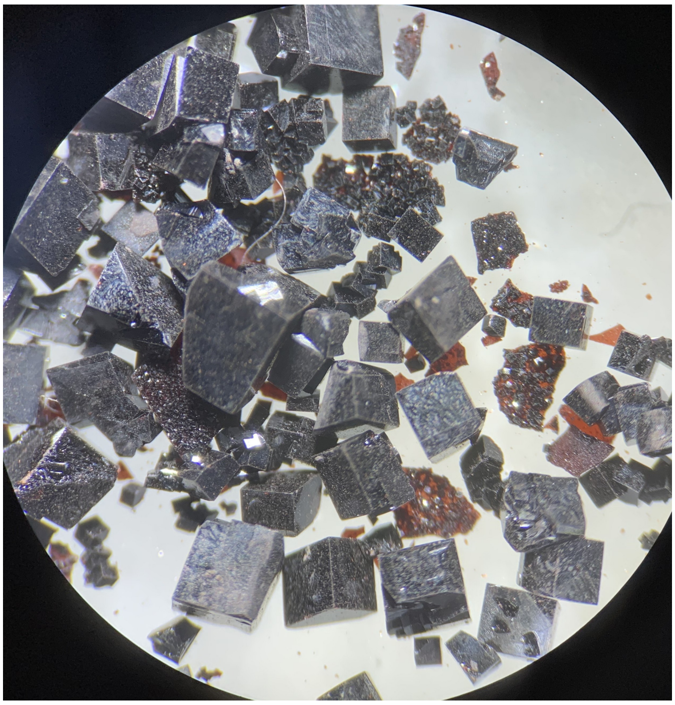

Figure S11: Brown cubic block shaped crystals of $(n\text{-}Bu_4N)_3MnV$ were used for all measurements.

S8 UV-VIS Spectroscopy

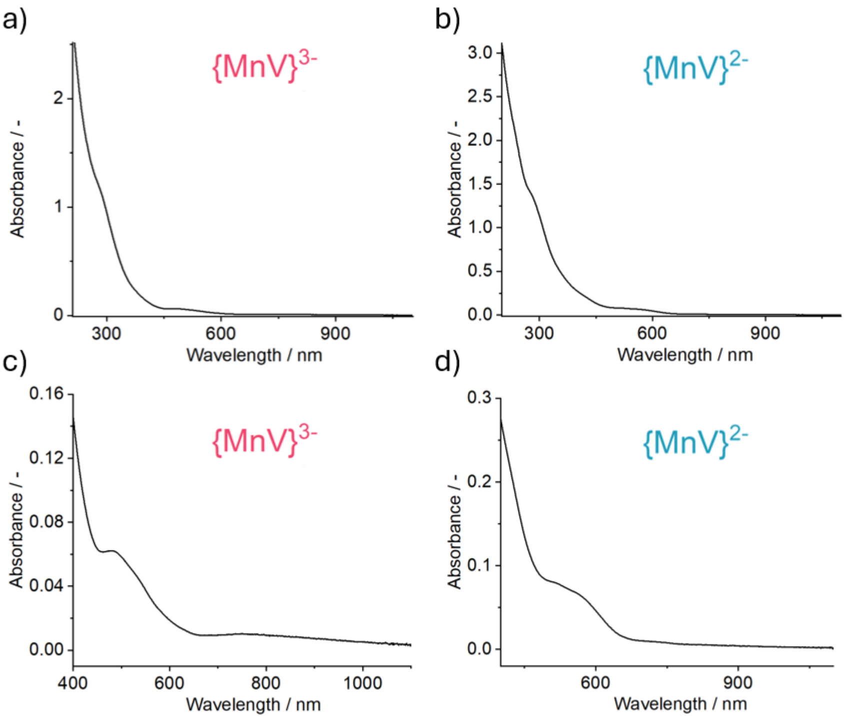

Figure S12: a, b) A UVVis spectrum of $\{MnV\}^{3-}$ and $\{MnV\}^{2-}$ was measured at ambient conditions at a concentration of 50 µM. The range of 400 nm to 1100 nm is shown in c) and d).

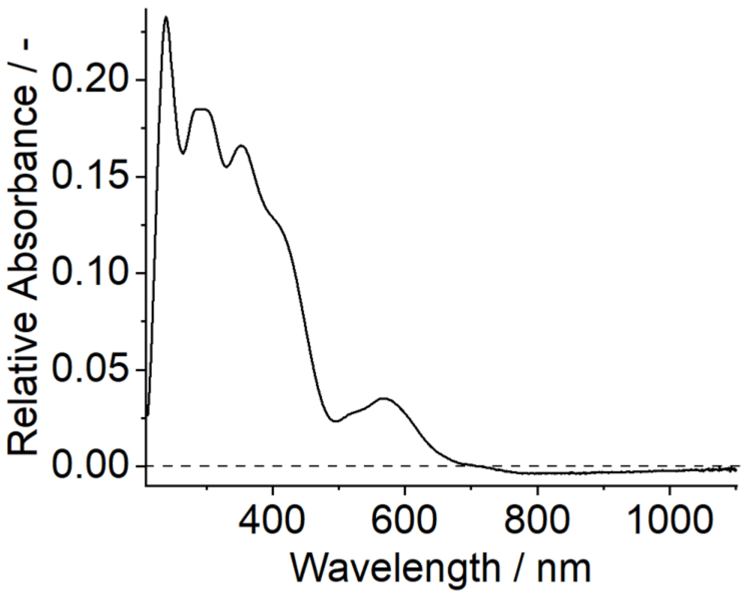

Figure S13: Relative absorbance of $\{MnV\}^{2-}$ compared to $\{MnV\}^{3-}$. The spectrum of $\{MnV\}^{3-}$ was subtracted as reference and agrees with the literature.[27]

S9 FTIR Spectroscopy

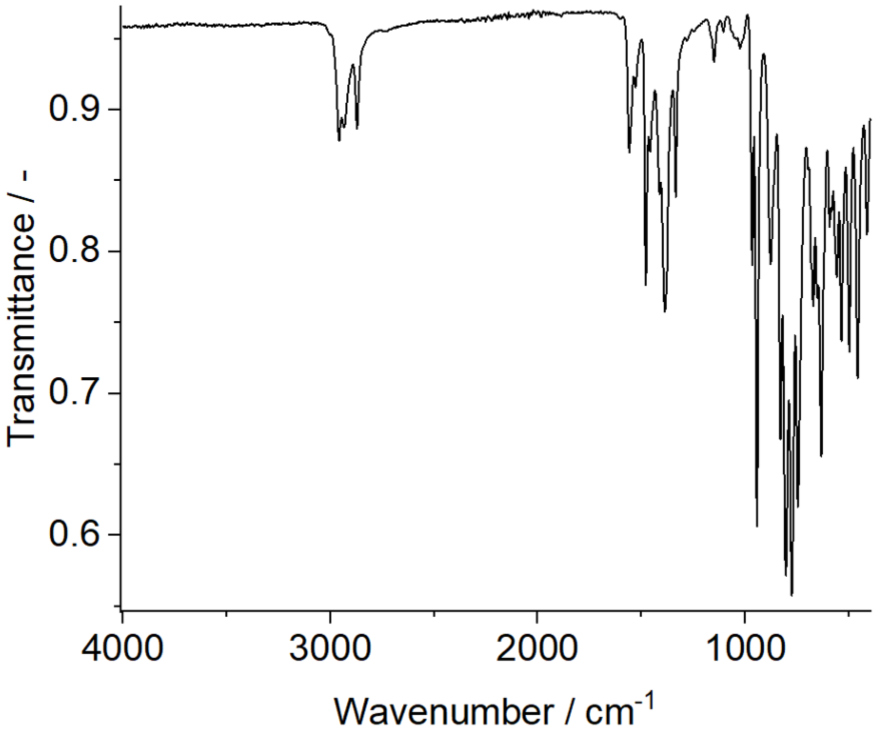

Figure S14: FTIR spectrum of (n-$Bu_4N)_3$MnV.

S10 Liquid IR Spectroscopy: Acetic Acid

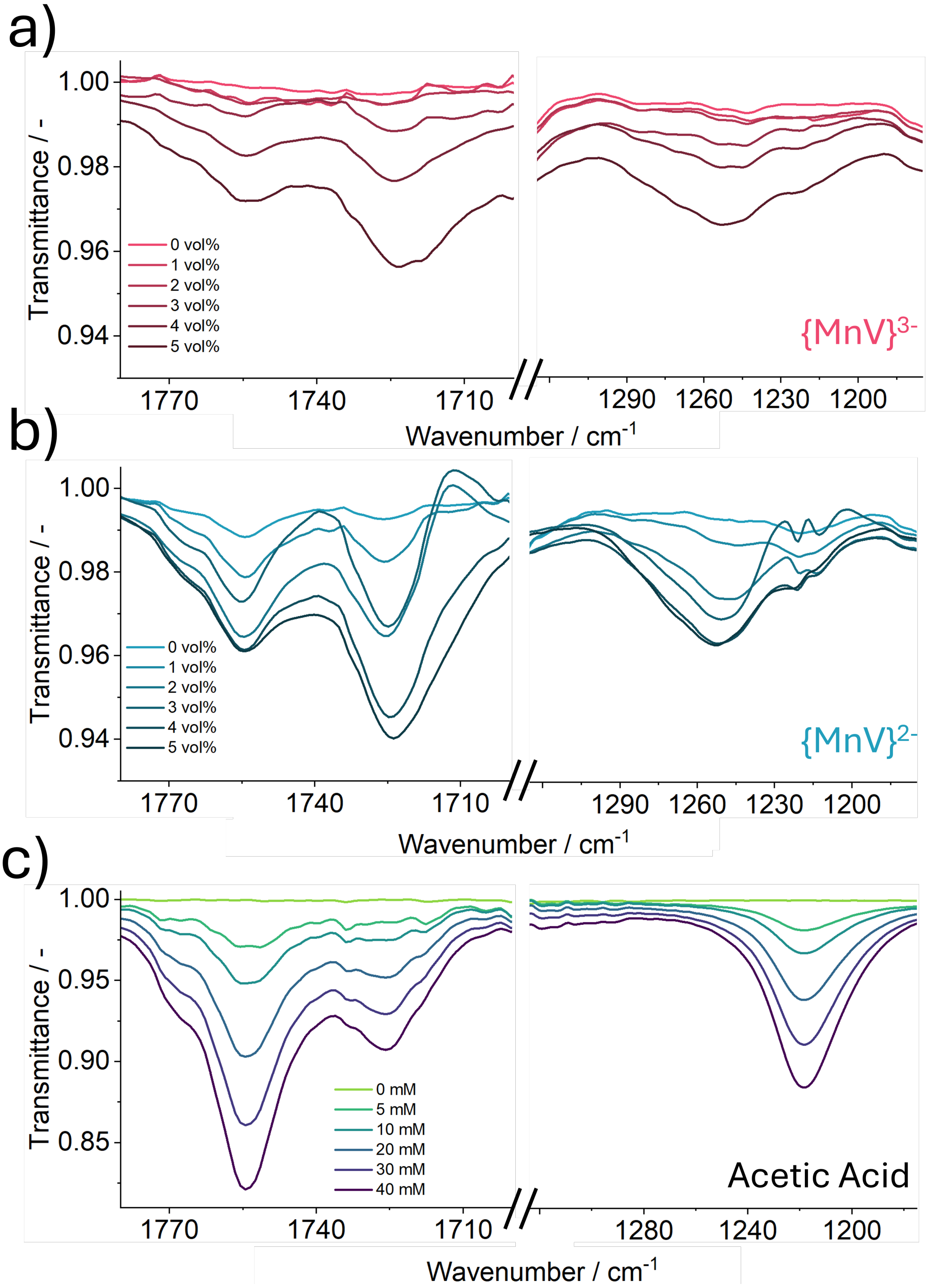

Figure S15: : Liquid FTIR signals of acetic acid in solutions of a) 10 mM $\{MnV\}^{3-}$ and b) $\{MnV\}^{2-}$ with increasing vol% of water at a pathlength of 0.05 mm. Acetic acid was measured at different concentrations in acetonitrile at a pathlength of 0.05 mm for quantification. The signal at 1218 cm^{1-} was used for linear regression.

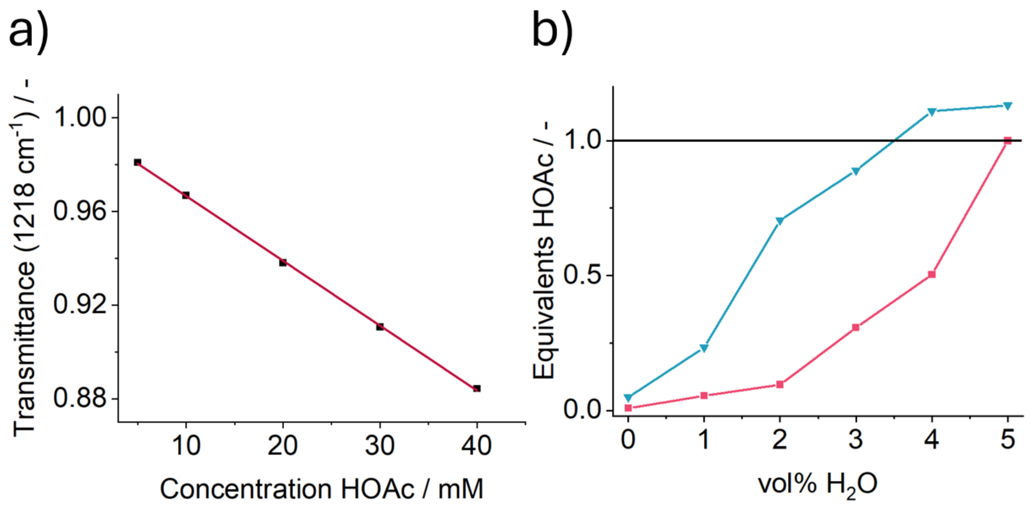

Figure S16: a) Linear regression of the signal intensity at 1218 cm^{-1} of acetic acid in acetonitrile and b) equivalents of free acetic acid depending on the water content. The signal of acetic acid increases faster for the samples of $\{MnV\}^{2-}$ compared to $\{MnV\}^{3-}$. The complex reaction mixture only allows for an estimation of the amount of free acetic acid, which reaches about 1 equivalent compared to the cluster concentration with increasing water content in agreement with the reaction pathway of ligand exchange presented in the literature.[28]

S11 ESI Mass Spectroscopy: $\{MnV\}^{3-}$ and $\{MnV\}^{2-}$

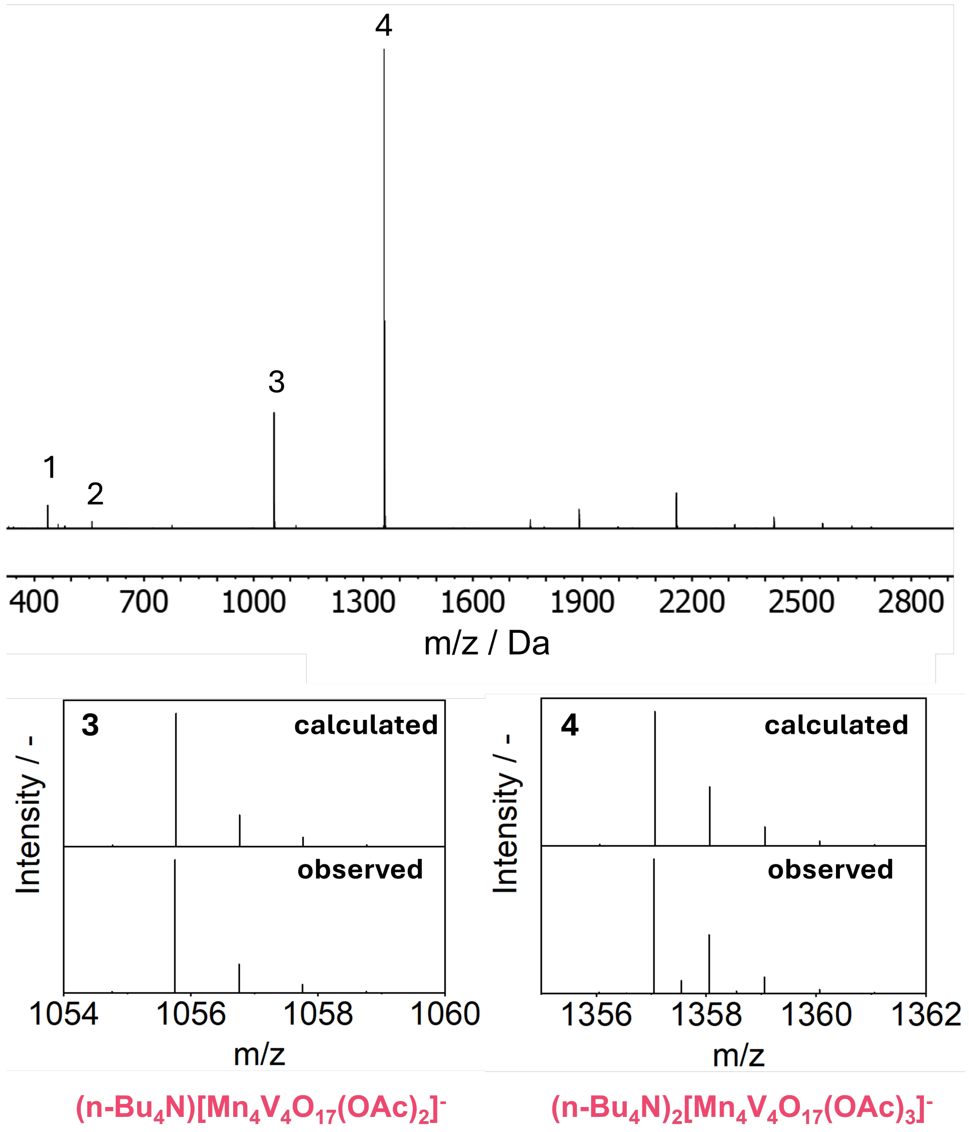

Figure S17: High-resolution negative-ion mode ESI mass spectrum of $\{MnV\}^{3-}$ (0.05mM) in acetonitrile. The isotopic distribution was calculated for the two main signals 3 and 4.

Table S13: Detailed peak assignment of the high-resolution negative-ion mode ESI mass spectrum of $\{MnV\}^{3-}$.

Nr.	Fragment	observed / m/z	calculated / m/z
1	$[Mn_4V_4O_{17}(OAc)_3]_2^-$	436.24	436,25
2	$(n\text{-}Bu_4N)[Mn_4V_4O_{17}(OAc)_3]_2^-$	557.38	557.39
3	**$(n\text{-}Bu_4N)[Mn_4V_4O_{17}(OAc)_2]^-$**	**1055.75**	**1055,77**
4	**$(n\text{-}Bu_4N)_2[Mn_4V_4O_{17}(OAc)_3]^-$**	**1357.05**	**1357.06**

Note that similar signals have been observed in previous literature.[26]

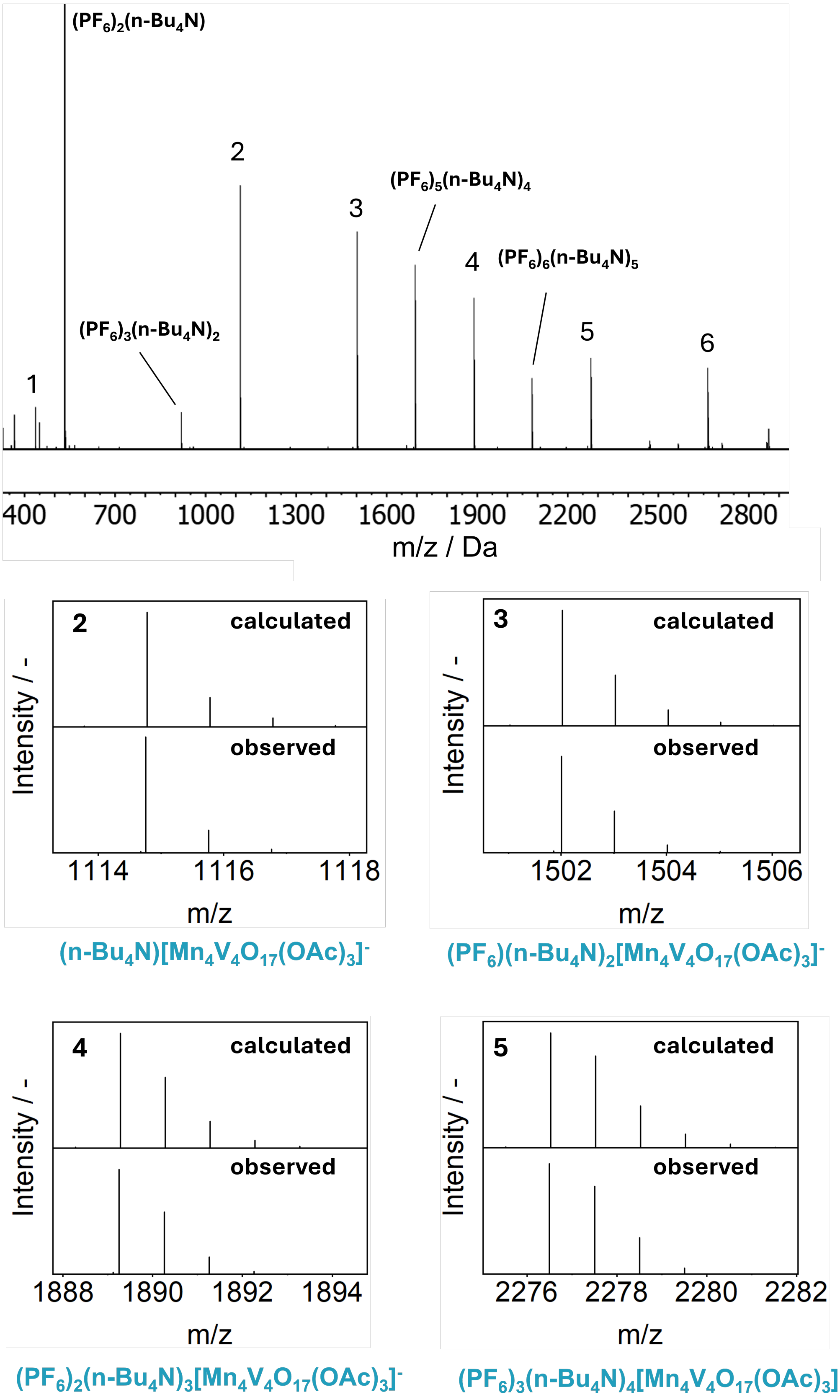

Figure S18: High-resolution negative-ion mode ESI mass spectrum of $\{MnV\}^{2-}$ (0.05mM) in acetonitrile containing residual (n-Bu_4N)PF_6 from the bulk electrolysis (5 mM).

Table S14: Peak assignments of the high-resolution negative-ion mode ESI mass spectrum of {MnV}$^{2-}$.

Nr.	Fragment	observed / m/z	calculated / m/z	
1	$[Mn_4V_4O_{17}(OAc)_3]_2^-$	436.24	436,25	
2	**$(n\text{-}Bu_4N)[Mn_4V_4O_{17}(OAc)_3]^-$**	**1114.76**	**1114.78**	
3	**$(PF_6)(n\text{-}Bu_4N)_2[Mn_4V_4O_{17}(OAc)_3]^-$**	**1502.01**	**1502.03**	
4	**$(PF_6)_2(n\text{-}Bu_4N)_3[Mn_4V_4O_{17}(OAc)_3]^-$**	**1889.26**	**1889.28**	
5	**$(PF_6)_3(n\text{-}Bu_4N)_4[Mn_4V_4O_{17}(OAc)_3]^-$**	**2276.50**	**2276.53**	
6	$(PF_6)_4(n\text{-}Bu_4N)_5[Mn_4V_4O_{17}(OAc)_3]^-$	2663.74	2663.77	

S12 ESI Mass Spectroscopy: Titration of H_2O

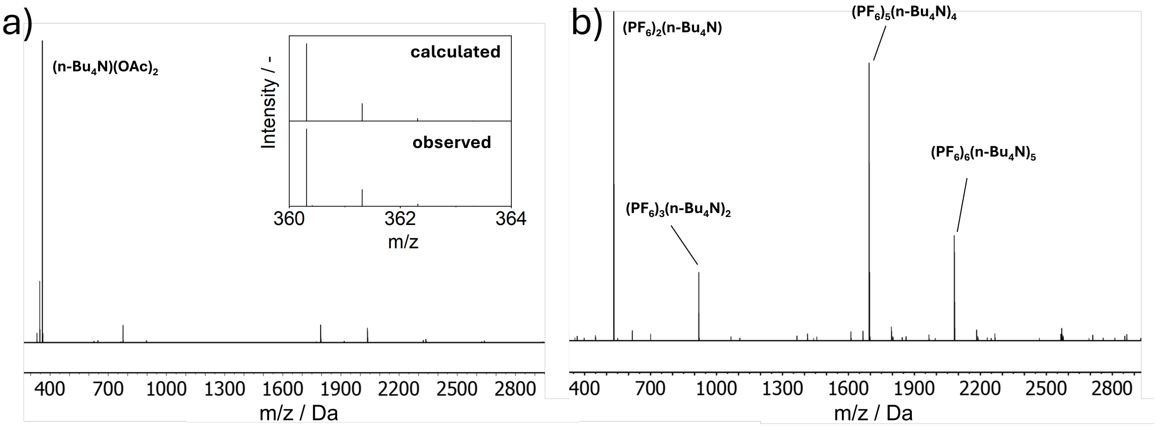

Figure S19: High-resolution negative-ion mode ESI mass spectrum of a) $\{MnV\}^{3-}$ including the assignment of $(n\text{-}Bu_4N)(OAc)_2{}^{-}$ and b) $\{MnV\}^{2-}$ (0.05mM) in acetonitrile containing 5 vol% water. In both spectra no cluster signals were detected likely due to ion pairing and aggregation.

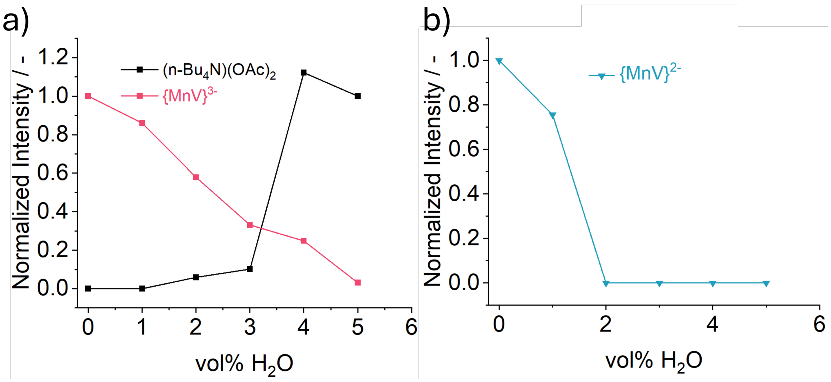

Figure S20: a) Normalized signal intensity of $(n\text{-}Bu_4N)(OAc)_2{}^{-}$ at increasing water content. The cluster signals of $\{MnV\}^{3-}$ decrease to a water content of 5 vol%. b) The cluster signals of $\{MnV\}^{2-}$ are not observable at a water content above 2 vol% likely due to aggregation. No acetate related signals were detected in the presence of electrolyte.

S13 Computed IR Spectra

IR spectra were computed using ORCA 6.0[?] at B3LYP/def2-SVP level of theory.[5–8] For systems treated with explicit solvation, a total of ten water molecules were placed around the catalyst.

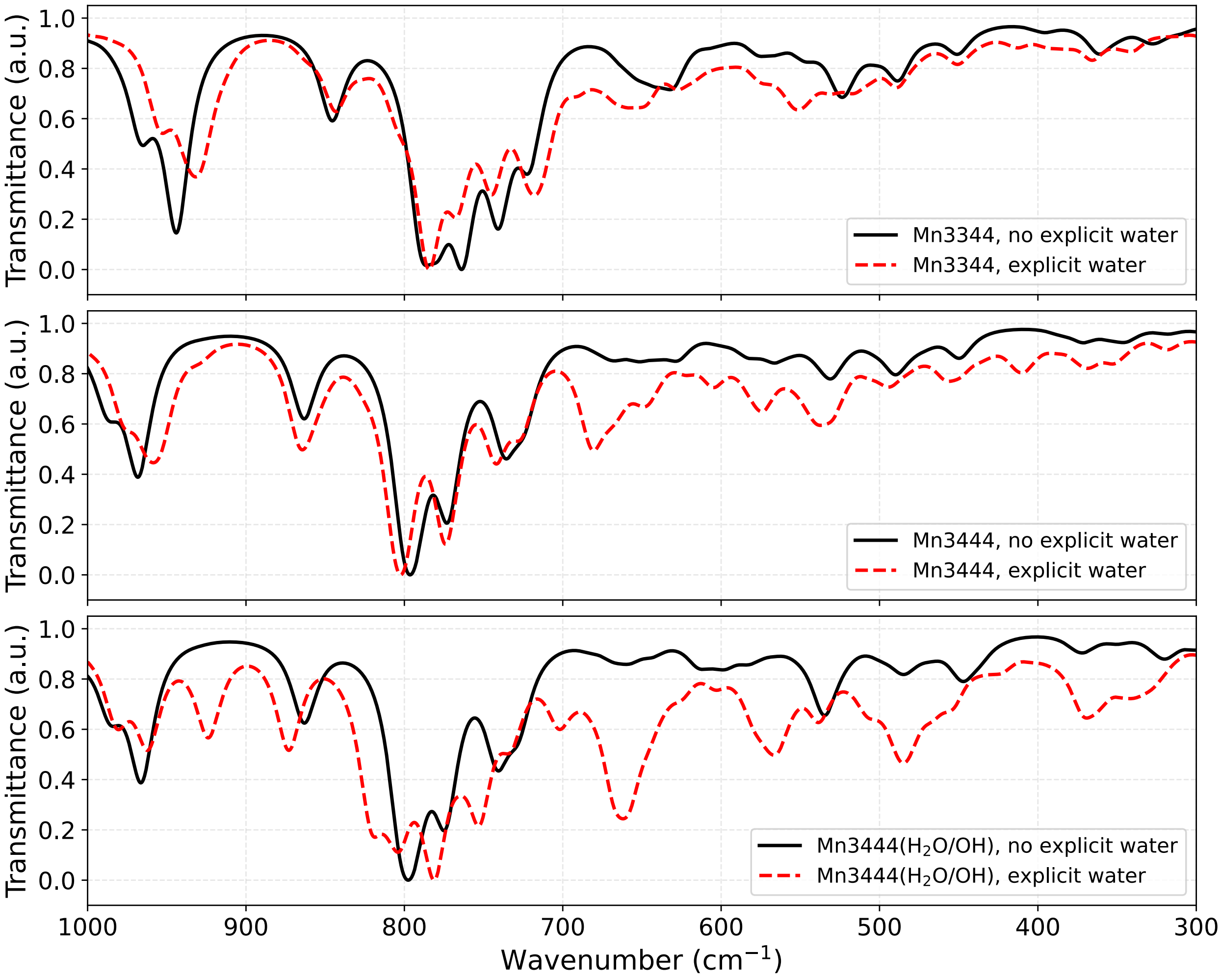

Figure S21: Simulated IR spectra for the {MnV}$^{3-}$ and {MnV}$^{2-}$ oxidation states. Black lines show systems without explicit water molecules around {MnV}$^{n-}$, while red dashed lines represent systems with explicit water included. For {MnV}$^{2-}$, an additional model was examined in which one acetate ligand was replaced by a water and an OH group.

S14 NMR Spectroscopy of $\{MnV\}^{3-}$: Peak Assignment

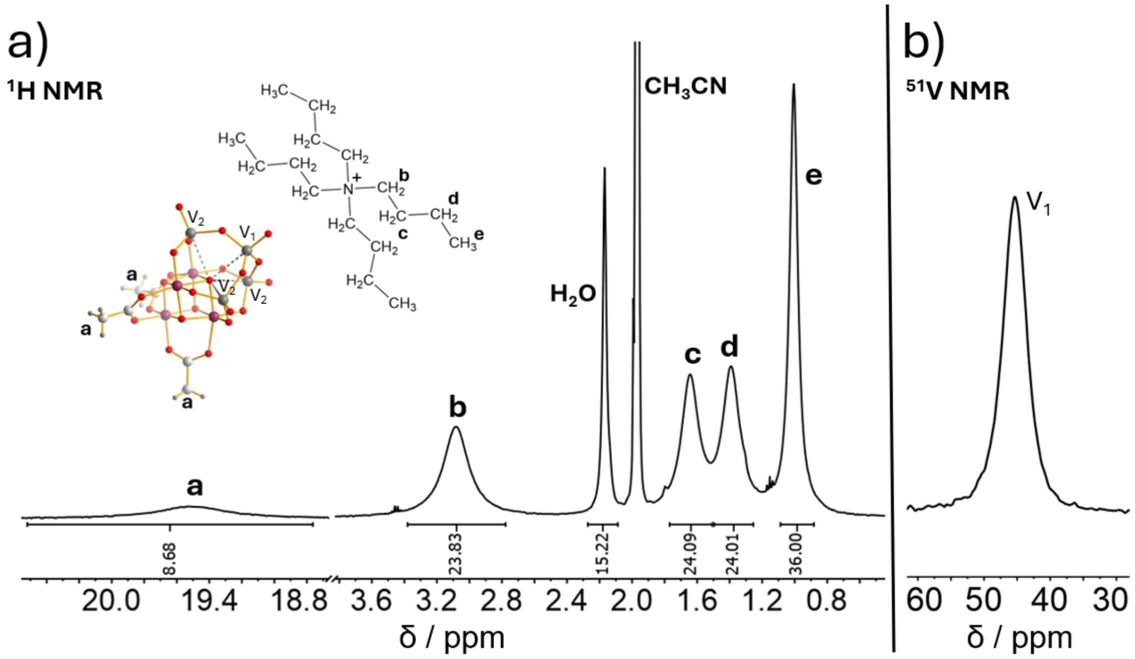

Figure S22: a) ^{1}H-NMR spectrum of 5 mM $\{MnV\}^{3-}$ solution in d3-CH_3CN with the resonances assigned. The sample was prepared under an inert atmosphere in the glove box. The integral of the water signal is slightly higher due to residual water present in the acetonitrile. The signal of the acetate ligand a is paramagnetically shifted to 19.5 ppm due to the proximity to the manganese centers. b) ^{51}V-NMR spectrum of 1 mM $\{MnV\}^{3-}$ solution in d3-CH_3CN. The ^{51}V V_1 resonance is paramagnetically shifted to +45 ppm. The V_2 signals are broadened beyond detection due to the close distance to the paramagnetic cubane core.

S15 NMR Spectroscopy: Titration with H_2O

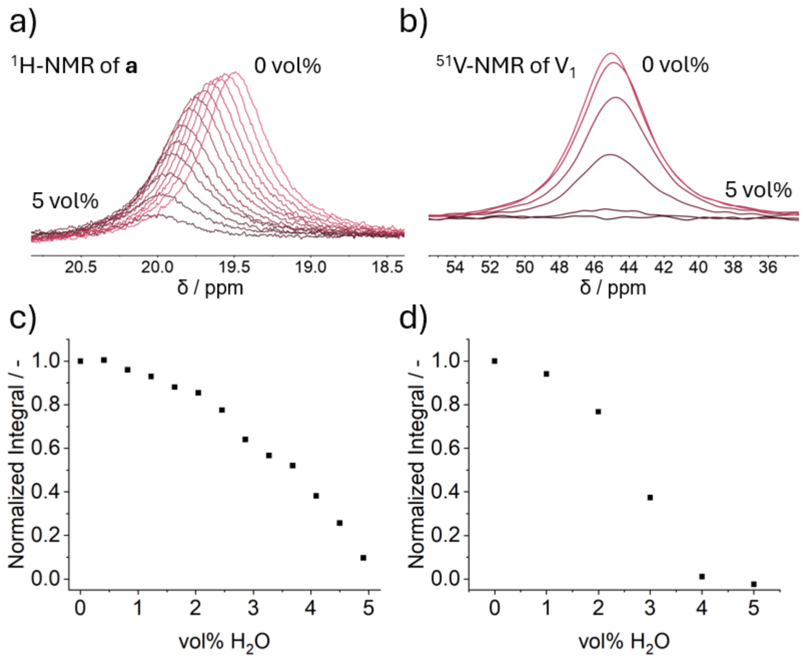

Figure S23: a): Overlaid ^{1}H NMR spectra of the $\{MnV\}^{3-}$ cluster in d3-CH_3CN increasing the water content with the spectral region between 18.0 ppm and 21.0 ppm magnified. The samples were prepared by adding 50 M solution of water in acetonitrile in 2.5 µL steps to a 0.5 mL of a 5 mM solution of $\{MnV\}^{3-}$ in d3-CH_3CN. Each sample was filled with d3-CH_3CN after to a total volume of 0.55 mL. b) ^{51}V-NMR spectrum of $\{MnV\}^{3-}$ at a concentration of 1 mM in deuterated acetonitrile with increasing water content. c) Normalized ^{1}H integral of the signal a as a function of the water content and d) The ^{51}V V_1 resonance becomes too broad to be detected probably due to aggregation or geometry changes by ligand exchange by higher water contents.

S16 ^{1}H-NMR Spectroscopy: T1 Measurements

The ^{1}H T1 relaxation time constants of the H_2O resonance of samples containing 5 mM $\{MnV\}^{3-}$ cluster in d3-CH_3CN increasing the amount of water were measured at ambient temperature using the inversion recovery sequence. To investigate the effect of the dissolved oxygen on the T1 relaxation two separate rows of T1 experiments (with dissolved oxygen and oxygen free) have been measured. The oxygen was removed by three freeze pump thaw cycles. Subsequently, the samples were sealed with Leica Criptoseal in the glovebox. As the main contribution to the T1 relaxation of all ^{1}H species in the solutions is the interaction with the paramagnetic cluster hardly any effect of the dissolved oxygen on the T1 relaxation time constants was detected.

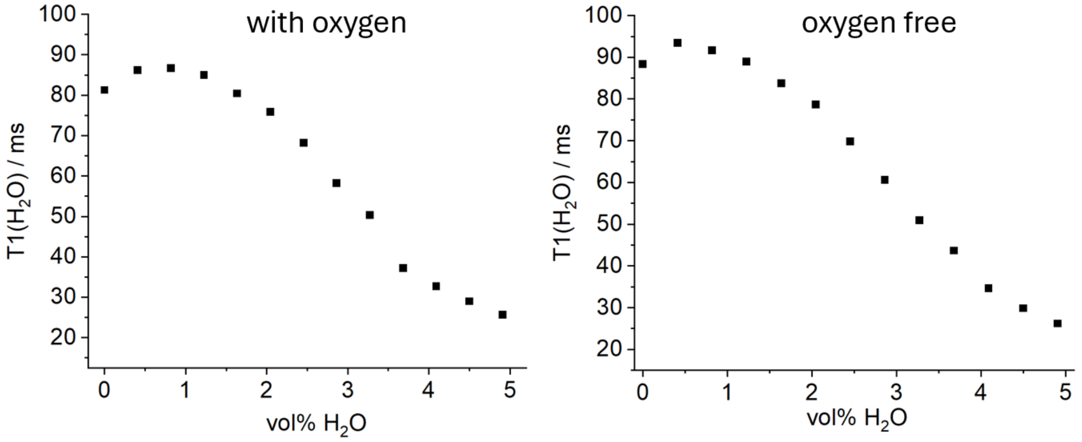

Figure S24: ^{1}H T1 relaxation time constants of the H_2O resonance of samples containing 5 mM $\{MnV\}^{3-}$ cluster in d3-CH_3CN increasing the water content measured with dissolved oxygen (left) and under oxygen free conditions (right) at ambient temperature. The influence of molecular paramagnetic oxygen on the T1 relaxation time constants is obscured by the stronger paramagnetic effect of the cluster.

S17 Electrochemistry: Electrochemical Generation of $\{MnV\}^{2-}$

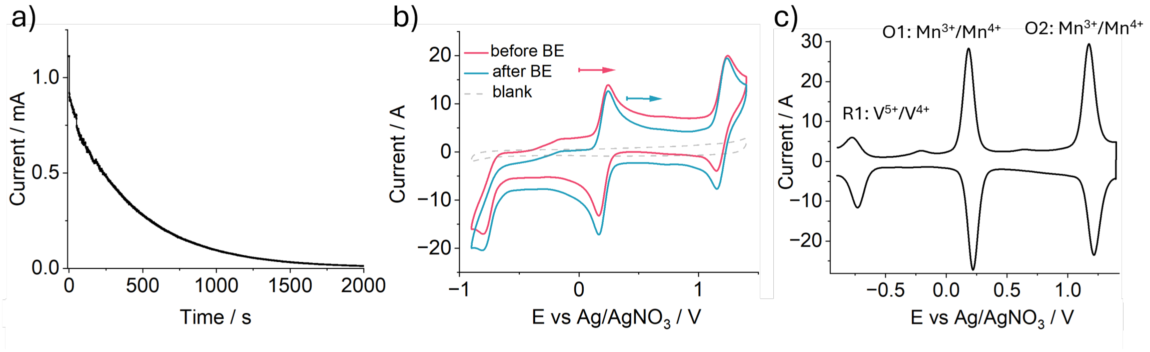

Figure S25: a) Chronoamperogram of bulk electrolysis (BE) at E = 0.65 V of 1 mM solution of $(n\text{-}Bu_4N)_3MnV$ in water-free acetonitrile containing 0.1 M nBu_4NPF_6 as supporting electrolyte. b) A cyclovoltammogram be-fore and after bulk electrolysis was recorded at a glassy carbon electrode (3 mm diam.) scan rate: 100 mV/s vs. $Ag/AgNO_3$. The open circuit potentials and scan directions are indicated by arrows. Note that no compound degradation is observed during the one-electron oxidation. c) The redox transitions were assigned using square wave voltammetry according to the literature.[26]

S18 Electrochemistry: Titration of H_2O

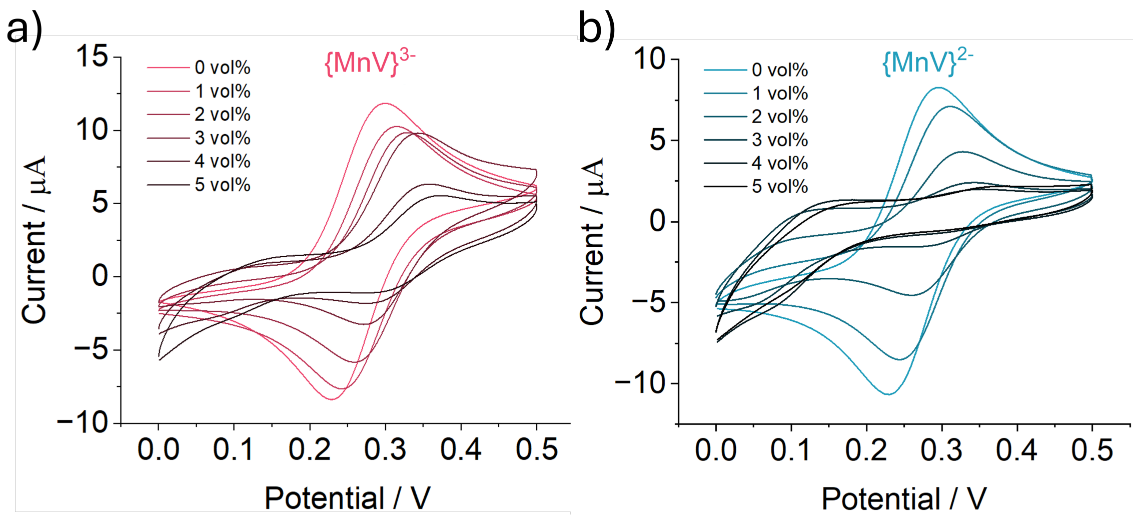

Figure S26: Cyclic voltammogram at increasing vol% water of a) $\{MnV\}^{3-}$ and b) $\{MnV\}^{2-}$ at a scan rate of 50 mV/s.

S19 Randles–Ševčík Analysis: Titration of H_2O

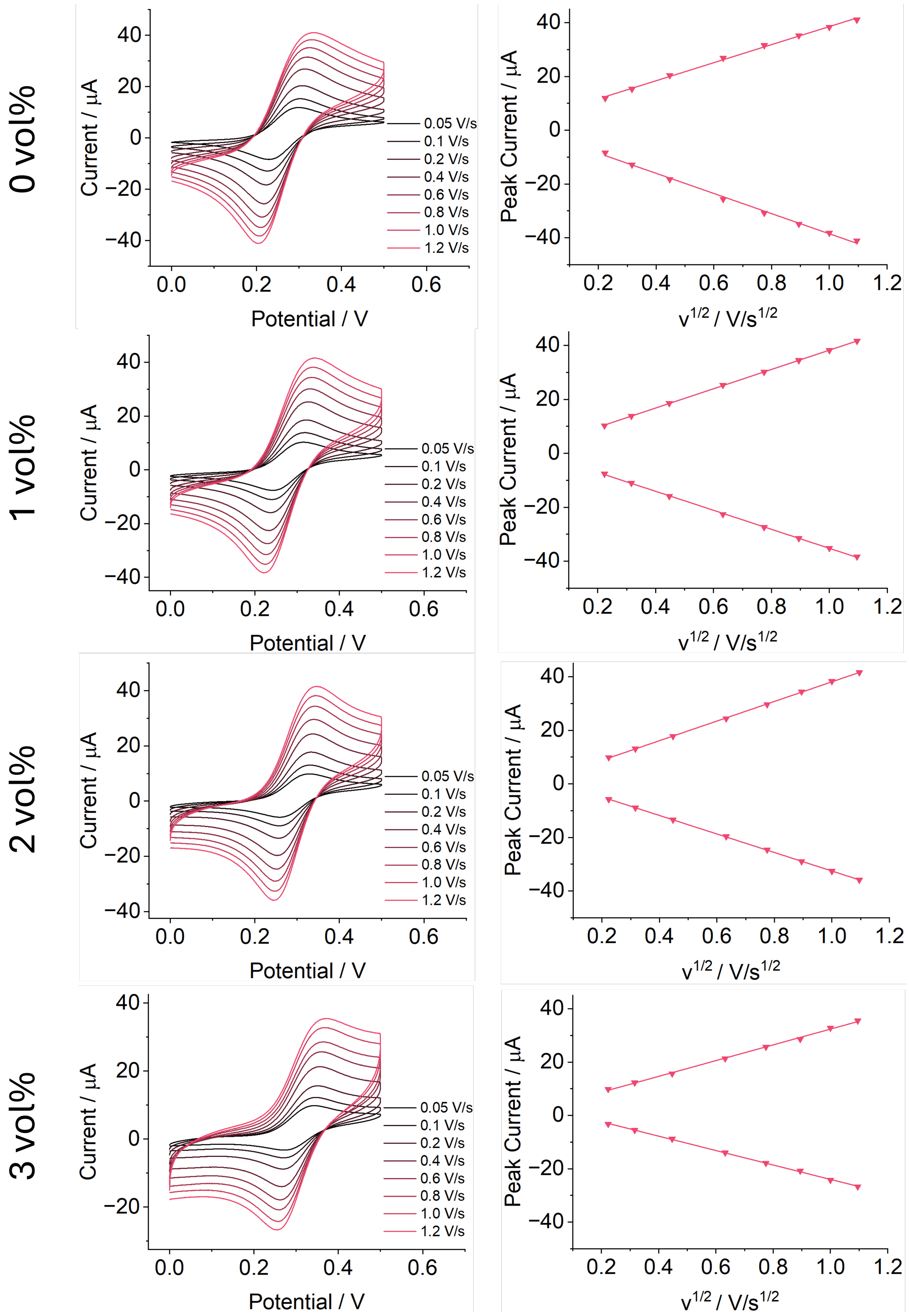

Figure S27: Randles–Ševčík analysis of 1 mM solution of {MnV}$^{3-}$ at different vol% of water.

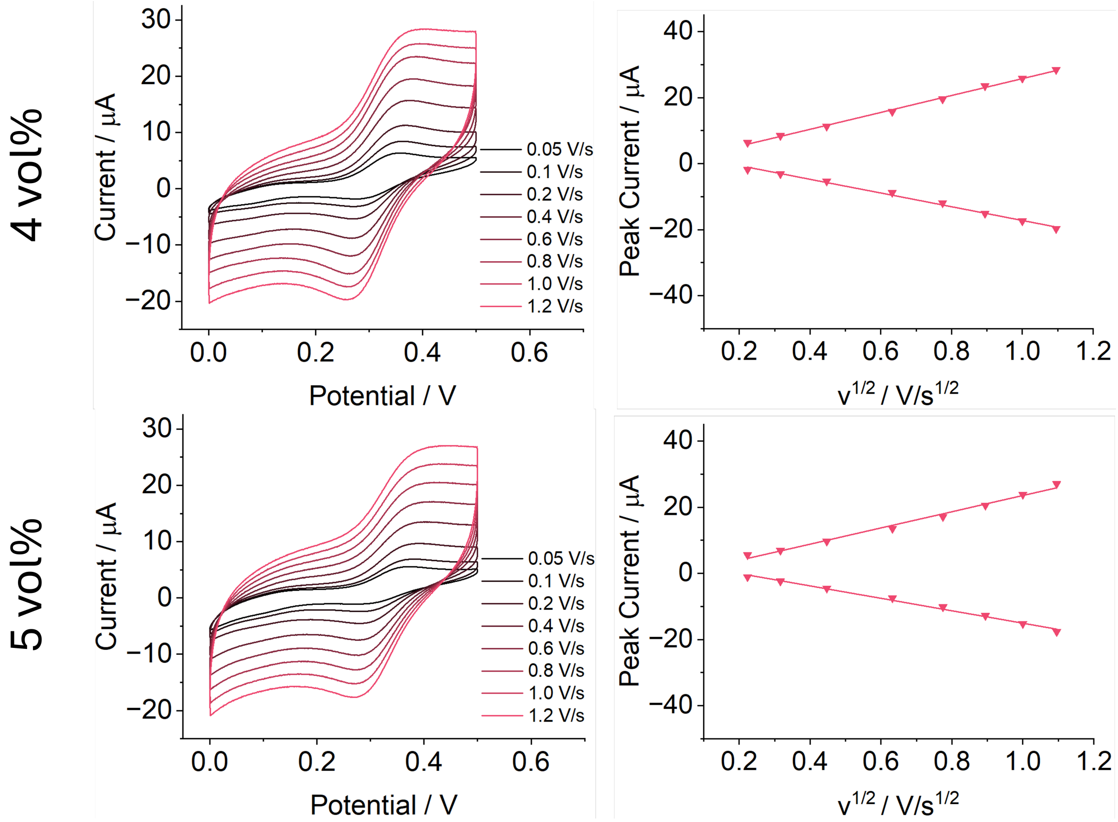

Figure S28: Randles–Ševčík analysis of 1 mM solution of $\{MnV\}^{3-}$ at different vol% of water.

Table S15: Slopes of the linear regression of anodic and cathodic peak currents i_{pa} and i_{pc} and average diffusion coefficients of $\{MnV\}^{3-}$determined by the Randles–Ševčík equation.

**vol% H2O**	**Slope i_{pa} [A $s^{1/2}V^{-1/2}$]**	**Slope ipc [A $s^{1/2}V^{-1/2}$]**	**Avg. Diffusion Coefficient [cm^2/s]**
0	3.36 x 10-5	-3.74 x 10-5	3.49 x 10-6
1	3.57 x 10-5	-3.52 x 10-5	3.48 x 10-6
2	3.66 x 10-5	-3.46 x 10-5	3.51 x 10-6
3	2.95 x 10-5	-2.71 x 10-5	2.22 x 10-6
4	2.55 x 10-5	-2.08 x 10-5	1.50 x 10-6
5	2.45 x 10-5	-1.88 x 10-5	1.32 x 10-6

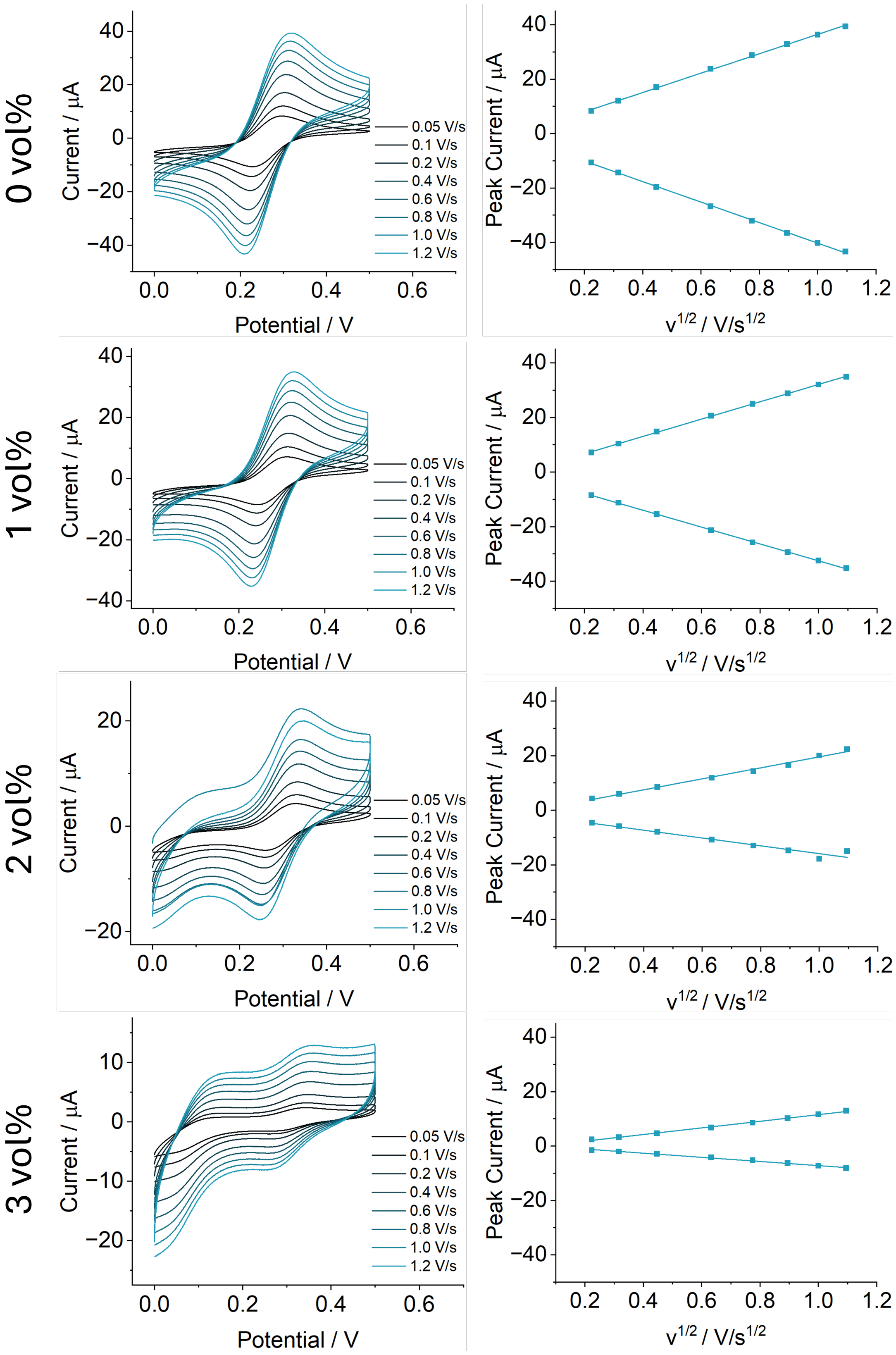

Figure S29: Randles–Ševčík analysis of 1 mM solution of $\{MnV\}^{2-}$ at different vol% of water.

Table S16: Slopes of the linear regression of anodic and cathodic peak currents i_{pa} and i_{pc} and average diffusion coefficients of $\{MnV\}^{2-}$ determined by the Randles–Ševčík equation.

vol% H2O	Slope i_{pa} [A $s^{1/2}V^{-1/2}$]	Slope ipc [A $s^{1/2}V^{-1/2}$]	Avg. Diffusion Coefficient [cm^2/s]
0	3.56 x 10-5	-3.77 x 10-5	3.76 x 10-6
1	3.17 x 10-5	-3.09 x 10-5	2.71 x 10-6
2	2.01 x 10-5	-1.43 x 10-5	0.84 x 10-6
3	1.22 x 10-5	-0.76 x 10-5	0.29 x 10-6

Supplementary References

[1] L. Martínez, R. Andrade, E. G. Birgin, J. M. Martínez, *Journal of Computational Chemistry* **2009**, *30*, 2157.

[2] C. I. Bayly, P. Cieplak, W. Cornell, P. A. Kollman, *The Journal of Physical Chemistry* **1993**, *97*, 10269.

[3] J. Wang, W. Wang, P. A. Kollman, D. A. Case, *Journal of Molecular Graphics and Modelling* **2006**, *25*, 247.

[4] J. Wang, R. M. Wolf, J. W. Caldwell, P. A. Kollman, D. A. Case, *Journal of Computational Chemistry* **2004**, *25*, 1157.

[5] J. P. Perdew, *Physical Review B* **1986**, *33*, 8822.

[6] C. Lee, W. Yang, R. G. Parr, *Physical Review B* **1988**, *37*, 785.

[7] F. Weigend, R. Ahlrichs, *Physical Chemistry Chemical Physics* **2005**, *7*, 3297.

[8] F. Weigend, *Physical Chemistry Chemical Physics* **2006**, *8*, 1057.

[9] S. Grimme, J. Antony, S. Ehrlich, H. Krieg, *The Journal of Chemical Physics* **2010**, *132*, 154104.

[10] S. Grimme, S. Ehrlich, L. Goerigk, *Journal of Computational Chemistry* **2011**, *32*, 1456.

[11] M. J. Frisch, G. W. Trucks, J. R. Cheeseman, G. Scalmani, M. Caricato, H. P. Hratchian, X. Li, V. Barone, J. Bloino, G. Zheng, T. Vreven, J. A. Montgomery, G. A. Petersson, G. E. Scuseria, H. B. Schlegel, H. Nakatsuji, A. F. Izmaylov, R. L. Martin, J. L. Sonnenberg, J. E. Peralta, J. J. Heyd, E. Brothers, F. Ogliaro, M. Bearpark, M. A. Robb, B. Mennucci, K. N. Kudin, V. N. Staroverov, R. Kobayashi, J. Normand, A. Rendell, R. Gomperts, V. G. Zakrzewski, M. Hada, M. Ehara, K. Toyota, R. Fukuda, J. Hasegawa, M. Ishida, T. Nakajima, Y. Honda, O. Kitao, H. Nakai, Gaussian 16, gaussian Inc. Wallingford CT 2016.

[12] A. M. Nikitin, A. P. Lyubartsev, *Journal of Computational Chemistry* **2007**, *28*, 2020.

[13] W. L. Jorgensen, J. Chandrasekhar, J. D. Madura, R. W. Impey, M. L. Klein, *The Journal of Chemical Physics* **1983**, *79*, 926.

[14] Y. Wu, H. L. Tepper, G. A. Voth, *The Journal of Chemical Physics* **2006**, *124*, 024503.

[15] F. Paesani, W. Zhang, D. A. Case, T. E. Cheatham, G. A. Voth, *The Journal of Chemical Physics* **2006**, *125*, 184507.

[16] A. W. Götz, M. J. Williamson, D. Xu, D. Poole, S. Le Grand, R. C. Walker, *Journal of Chemical Theory and Computation* **2012**, *8*, 1542.

[17] R. Salomon-Ferrer, A. W. Götz, D. Poole, S. Le Grand, R. C. Walker, *Journal of Chemical Theory and Computation* **2013**, *9*, 3878.

[18] J.-P. Ryckaert, G. Ciccotti, H. J. Berendsen, *Journal of Computational Physics* **1977**, *23*, 327.

[19] D. R. Roe, T. E. Cheatham, *Journal of Chemical Theory and Computation* **2013**, *9*, 3084.

[20] S. Polonius, D. Lehrner, L. González, S. Mai, *Journal of Chemical Theory and Computation* **2024**, *20*, 4738.

[21] G. Cárdenas, P. Marquetand, S. Mai, L. González, *Catalysts* **2021**, *11*, 493.

[22] H. French, *The Journal of Chemical Thermodynamics* **1987**, *19*, 1155.

[23] S. Sultana, M. N. Ullah, S. K. Begum, *Chemical Physics Impact* **2025**, *10*, 100855.

[24] S. Tippner, P. Lechner, L. González, S. Mai, *The Journal of Chemical Physics* **2024**, *160*, 084306.

[25] W. Humphrey, A. Dalke, K. Schulten, *Journal of Molecular Graphics* **1996**, *14*, 33.

[26] B. Schwarz, J. Forster, M. K. Goetz, D. Yücel, C. Berger, T. Jacob, C. Streb, *Angewandte Chemie International Edition* **2016**, *55*, 6329.

[27] S. Mai, S. Klingler, I. Trentin, J. Kund, M. Holzer, A. Andreeva, R. Stach, C. Kranz, C. Streb, B. Mizaikoff, L. González, *Chemistry – A European Journal* **2021**, *27*, 17078.

[28] G. Cárdenas, I. Trentin, L. Schwiedrzik, D. Hernández-Castillo, G. A. Lowe, J. Kund, C. Kranz, S. Klingler, R. Stach, B. Mizaikoff, P. Marquetand, J. J. Nogueira, C. Streb, L. González, *Chemical Science* **2021**, *12*, 12918.
